# Rapid Monitoring and Quantification of Primary and Secondary Oxidative Markers in Edible Oils During Deep Frying Using Near-Infrared Spectroscopy and Chemometrics

**DOI:** 10.3390/foods15030557

**Published:** 2026-02-04

**Authors:** Taha Mehany, José M. González-Sáiz, Consuelo Pizarro

**Affiliations:** Department of Chemistry, University of La Rioja, 26006 Logroño, Spain; taha.abdellatif@unirioja.es (T.M.); josemaria.gonzalez@unirioja.es (J.M.G.-S.)

**Keywords:** hydroxytyrosol, medicinal plants, bioactive compounds, near-infrared spectroscopy, chemometrics, oxidative stability, polyphenols, vegetable oils, SELECT algorithm, TOTOX index

## Abstract

Background: Oxidative degradation during deep frying negatively affects the nutritional quality and stability of edible oils. Rapid, non-destructive methods to monitor oxidation, particularly in antioxidant-enriched oils, are therefore of growing interest. Materials and Methods: This study investigates the potential of near-infrared (NIR) spectroscopy combined with chemometric modeling—specifically the Stepwise Decorrelation of Variables (SELECT) algorithm and Ordinary Least Squares (OLS) regression—to quantitatively assess oxidation dynamics in edible oils enriched with hydroxytyrosol extract from olive fruit during deep frying. Extra virgin olive oil, virgin olive oil, refined olive oil, refined sunflower oil, and high-oleic sunflower oil were evaluated under controlled thermal degradation conditions. Results: Variable selection identified key NIR spectral regions related to acidity, conjugated dienes (K_232_), secondary oxidation indices (K_270_ and ΔK), peroxide value (PV), anisidine value (AnV), and the total oxidation (TOTOX) index. From 700 measured wavelengths, a limited number were sufficient for robust prediction (16–30 wavelengths depending on the parameter), with critical sensitivity observed around 1792 nm and 1392 nm. The optimized NIR–SELECT–OLS models showed strong predictive performance across oil types (R^2^ > 0.90; explained variance > 85%). Conclusions: The results demonstrate that hydroxytyrosol enrichment enhances the oxidative and nutritional stability of edible oils during deep frying. Moreover, the integration of NIR spectroscopy with chemometric modeling provides an effective, non-destructive tool for real-time monitoring of oil oxidation, supporting sustainable quality control, process optimization, and antioxidant fortification in functional edible oils.

## 1. Introduction

Extra virgin olive oil (EVOO) has been used since ancient times as both a seasoning and cooking ingredient. In recent decades, it has gained recognition as a key component of the Mediterranean diet, thanks to growing evidence of its nutritional and health benefits. EVOO is extracted mechanically from olives without chemicals, and its quality depends on the olive variety, environment, and production process. It is rich in monounsaturated fats, especially oleic acid, and contains small but powerful bioactive compounds such as polyphenols, phytosterols, and squalene. These contribute to its distinct taste and are largely responsible for its antioxidant and anti-inflammatory properties. Regular consumption of EVOO is associated with reduced risk of cardiovascular disease, cancer, neurodegenerative disorders, and other chronic illnesses [1,2,3,4]. 

Near-infrared (NIR) spectroscopy has recently been utilized to monitor, qualify, and quantify the chemical composition and oxidation progress of edible oils [5,6]. The technique’s non-destructive nature and rapid analysis capabilities make it particularly attractive for industrial applications, where multiple quality parameters can be determined in a single measurement without requiring chemical reagents or extensive sample preparation [7]. The effectiveness of NIR spectroscopy extends beyond basic quality parameters to include fatty acid content prediction and nutritional labeling applications. Studies have demonstrated R^2^CV values of up to 0.88 for various oil quality indicators [8]. Comparative analyses have further shown that NIR spectroscopy consistently outperforms UV–Vis spectroscopy, providing lower root mean square error values and higher correlation coefficients, thereby reinforcing its suitability for rapid oil quality assessment.

Recently, NIR spectroscopy has emerged as a powerful, rapid, and non-destructive analytical technique for evaluating the quality and oxidative status of edible oils, offering a sustainable alternative to conventional wet-chemical methods that are time-consuming, costly, and generate chemical waste. Extensive research has demonstrated the strong potential of NIR spectroscopy, coupled with chemometric modeling, for the quantification of both primary and secondary oxidative markers in olive oil and related lipid matrices. In particular, key quality parameters associated with primary oxidation, such as peroxide value (PV) and conjugated dienes (K_232_), as well as secondary oxidation indicators including conjugated trienes (K_270_) and free acidity, have been successfully predicted with high accuracy using NIR-based models. García Martín [9] comprehensively reviewed the state of the art in olive oil analysis and concluded that free acidity, PV, K_232_, and K_270_—recognized as the most relevant oxidative quality indices—can be reliably quantified by NIR spectroscopy with high precision. Similarly, Cayuela Sánchez et al. [10] demonstrated that visible/NIR spectroscopy enables the rapid, multiparametric determination of oxidative stability and major oxidation-related quality indices, including free fatty acids, PV, and conjugated dienes, with robust predictive performance. Collectively, these studies confirm the suitability of NIR spectroscopy for monitoring both early-stage lipid oxidation (primary oxidation products) and advanced degradation processes (secondary oxidation products).

Despite these advances, most existing studies have focused primarily on olive oil under standard storage or mild processing conditions. Comparatively little attention has been given to complex thermal processes such as deep frying, where oxidation pathways are accelerated and evolve dynamically, or to the combined effect of thermal degradation and antioxidant fortification. Moreover, the simultaneous prediction of a comprehensive set of primary and secondary oxidation markers across different oil matrices remains insufficiently explored. Consequently, there is a clear need to extend NIR-based methodologies to thermally stressed and functionally enriched oils to enable real-time, multiparametric oxidation assessment under realistic processing conditions.

Recent advances have incorporated deep learning approaches and domain adaptation techniques to further enhance spectroscopic analysis, particularly for small datasets common in specialized oil analysis applications [11]. These developments, together with the increasing availability of portable and low-cost spectroscopic instruments, have made NIR-based methods more accessible while maintaining acceptable levels of accuracy, thereby lowering the barrier to adoption for oil producers and quality control laboratories [7,12].

Furthermore, several studies have investigated the use of low-cost instruments to evaluate sensory attributes and bioactive compounds in extra virgin olive oil (EVOO), virgin olive oil (VOO), and refined olive oil, aiming to facilitate rapid screening and classification methods for producers [13,14]. However, to the best of our knowledge, no comprehensive study has yet integrated NIR spectroscopy with multivariate analysis to simultaneously quantify both primary and secondary oxidation markers across EVOO, VOO, refined sunflower oil, and high-oleic sunflower oil under deep frying conditions and antioxidant supplementation.

Addressing this gap represents the central novelty of the present work. This study quantitatively evaluates key primary (peroxide value, conjugated dienes) and secondary (anisidine value, K_270_, ΔK) oxidation markers, along with acidity and the total oxidation (TOTOX) index, across multiple oil types subjected to thermal degradation and enrichment with a natural olive-derived extract. By simultaneously considering oil type, frying-induced oxidation, and antioxidant fortification, this work provides a more holistic and application-oriented assessment of oxidation dynamics than previously reported.

To achieve this, the SELECT algorithm was applied to the NIR spectral data to identify a compact yet highly informative subset of predictor variables. SELECT was chosen because it is a variable selection method rather than a regression technique, in contrast to Partial Least Squares (PLS), which builds latent variables from the entire spectral matrix. The key mathematical advantage of SELECT lies in its stepwise decorrelation strategy, which iteratively selects wavelengths that provide unique, non-redundant information, thereby minimizing multicollinearity and enhancing model interpretability—an important benefit for spectroscopic applications where strong variable correlation is common. This approach allows direct association between selected wavelengths and underlying chemical features, which is often less straightforward in PLS-based models. Following variable selection, ordinary least squares (OLS) regression was employed using the selected wavelengths to construct robust, transparent, and easily interpretable predictive models [15,16,17]. Overall, this study demonstrates the novel integration of NIR spectroscopy, SELECT-based variable selection, and OLS regression for real-time oxidation profiling and quality monitoring in thermally stressed, antioxidant-enriched edible oils, offering a practical and scalable tool for both industrial and research applications.

## 2. Materials and Methods

### 2.1. Materials and Samples

Olive fruit dry extract (20% hydroxytyrosol) was obtained from Natac BioTech (Natac, HQ-Europe, Alcorcon, Madrid, Spain). Ethanol (≥99.93% purity), chloroform (≥99% purity), sodium hydroxide (NaOH, 0.1 N), and cyclohexane (≥99.8% purity) were obtained from VWR International (Fontenay-sous-Bois Cedex, France). Glacial acetic acid (≥99% purity) was procured from Fisher Scientific Ltd. (Loughborough, UK). Diethyl ether (≥99.8% purity) was supplied by Honeywell Riedel-de Haen GmbH (Seelze, Germany), while isooctane (2,2,4-trimethylpentane, ≥99.8% purity) was purchased from Scharlab S.L. (Barcelona, Spain). Additionally, potassium iodide (≥99.0% purity), p-anisidine (99% purity), and sodium thiosulfate pentahydrate (Na_2_S_2_O_3_·5H_2_O, 99% purity) were obtained from Sigma-Aldrich (St. Louis, MO, USA).

This study examined a range of vegetable oils sourced from local Spanish suppliers. The collection included nine types of EVOO derived from different cultivars—Picual, Cornicabra, Empeltre, Arbequina, Hojiblanca, Manzanilla Cacereña, Royuela/Arróniz, Koroneiki, and Arbosana. Additionally, the sample set comprised one EVOO blended with refined olive oil (ROO), referred to as “olive oil 1°”; one pomace olive oil (a blend of refined pomace olive oil and EVOO, commonly known as orujo oil in Spain); and one virgin olive oil mixed with ROO, labeled “olive oil 0.4°”. Refined sunflower oil and refined high-oleic sunflower oil were also included in the analysis.

A total of 11 samples were evaluated from each olive oil category, including both control samples (non-fried and/or unsupplemented oils) and 8 samples subjected to deep frying under varying conditions. These conditions included different frying times, temperatures, and levels of polyphenol supplementation. Specifically, Experiments 1–4 involved non-supplemented samples, while Experiments 5–8 involved polyphenol-supplemented samples.

In addition, 5 samples were analyzed from each sunflower oil category. Overall, 142 oil samples were studied, representing a comprehensive range of oil types, treatment conditions, and controls.

For each sample, standard reference measurements were performed using UV–Vis spectrophotometry and titration methods to determine key quality and oxidation parameters such as acidity, PV, AnV, TOTOX index, and specific extinction coefficients (K-values). All analyses were conducted in triplicate. In total, 426 NIR spectra were acquired, enabling a rapid, non-destructive, and sustainable approach to oil quality assessment.

### 2.2. Exogenous Polyphenol Supplementation of Olive Oil

To improve the functional qualities of olive oils, they were fortified with a hydroxytyrosol-rich extract derived from olive fruit extract (OFE). This enrichment process involved adding OFE to different types of olive oils—extra virgin, virgin, or refined—referred to as Control 1. The result was a polyphenol-enhanced oil, which was then blended back with the original oil to produce Control 2. Control 2 was prepared following the method of Mehany et al. [18]. Briefly, 100 g of OFE was dissolved in 1000 g of water (10% *w*/*w*) and mechanically stirred for 30 min. The solution was then mixed with olive oil at a ratio of 200 g:500 g (aqueous:olive oil, *w*/*w*) and stirred for 1 h at room temperature using an IKA-WERKE magnetic stirrer (Staufen, Germany). The resulting mixture was centrifuged at 9961× *g* for 20 min using a Sorvall RC-6 Plus centrifuge (Osterode, Germany). The final product was stored in amber-colored containers at 7 ± 2 °C until further analysis.

### 2.3. Deep Frying

Different categories of olive and sunflower oils were exposed to thermal treatment using a 0.5 L flask and a Soxhlet-type heating device (SELECTA, Barcelona, Spain). In each deep frying trial, 0.4 L of oil was heated at 170 ± 10 °C and 210 ± 10 °C for 3 and 6 h. After heating, 400 mL of oil was collected into amber glass containers to examine oxidative degradation under varying conditions—oil type, frying temperature and duration, and the presence of natural antioxidants. Samples were stored in the dark at 5 °C to prevent further oxidation. For each sample, the oxidative stability was assessed through indicators of primary oxidation and secondary oxidation. In addition, NIR spectroscopy was performed on both untreated (control) and thermally processed oils across all categories.

### 2.4. Conventional Analysis of Oxidation Markers

The primary and secondary oxidation status of the oils was assessed using established analytical methods. Free acidity measures the concentration of free fatty acids released from triglycerides due to hydrolysis, determined by titration with a standardized base (e.g., NaOH), where the amount of base required reflects the acidity. Peroxide value quantifies peroxides and hydroperoxides, the primary oxidation products, by reacting the oil with potassium iodide to liberate iodine, which is then titrated with sodium thiosulfate. Extinction coefficients (K_232_ and K_270_) measure UV absorbance at 232 nm and 270 nm, corresponding to conjugated dienes (K_232_) and conjugated trienes or secondary oxidation products (K_270_), providing an estimate of lipid oxidation according to Beer–Lambert law. Finally, ΔK evaluates subtle deviations from theoretical absorbance at specific UV wavelengths, allowing detection of advanced oxidation or oil degradation not captured by K_232_ or K_270_ alone.

A conventional analysis was conducted to assess oxidation markers in the deep-fried oil samples. Primary and secondary oxidation indicators—including free acidity (expressed as % oleic acid), peroxide value (PV), and UV–Vis absorbance characteristics—were evaluated using a UV–Vis spectrophotometer (Model 8453, Hewlett Packard, Waldbronn, Germany) at 232 nm, 270 nm, and for ΔK values [19]. The anisidine value, representing secondary oxidation products, was determined following the AOCS official method [20].

### 2.5. TOTOX

The total oxidation value (TOTOX) of the edible oil samples was calculated to estimate the overall oxidative degradation [21], using the following equation:

TOTOX = 2 × PV + AnV(1)

where PV represents the peroxide value (indicating primary oxidation products) and AnV denotes the anisidine value (reflecting secondary oxidation compounds).

### 2.6. NIR Spectra Acquisition

A total of 426 spectra were recorded from 142 oil samples (Figure S1), each analyzed in triplicate using NIR spectroscopy. Prior to spectral measurement, the samples underwent centrifugation at 20,000 rpm for 30 min (using a Sorvall RC-6 Plus, Dreieich, Germany) to remove particles and dispersed water droplets, reducing light scattering effects. The NIR spectra were acquired using a Foss NIRSystems 5000 spectrophotometer (Foss NIRSystems, Silver Spring, MD, USA), which included a thermostated liquid analyzer module and a Suprasil quartz flow cell. The acquisition parameters were set to a 10 mm optical path length, covering a wavelength range from 1100 to 2498 nm, with a spectral resolution of 2 nm and 32 scans per spectrum.

### 2.7. Data Analyses and Chemometrics

Conventional analytical results were expressed as mean ± standard deviation (SD) using SPSS software (version 28, IBM, Chicago, IL, USA); these data were visualized by Origin 2025 software (OriginLab, Northampton, MA, USA). Further, these means were used for subsequent multivariate analyses. Parameters such as acidity, peroxide value, spectrophotometric indices, anisidine value, and total oxidation (TOTOX) in various olive oil samples (non-fried, fried, supplemented, and un-supplemented), as well as sunflower and high-oleic sunflower oils, were modeled using multivariate techniques.

NIR spectral data were analyzed using V-PARVUS software (PARVUS2011, Michele Forina, Genoa, Italy). Variable selection was performed with the SELECT algorithm, followed by ordinary least squares regression (SELECT-OLS) based on the selected spectral variables. To enhance model performance, autoscaling preprocessing was applied. SELECT prioritizes predictors based on their correlation with the response variable and eliminates redundant variables through iterative decorrelation. This improves both model interpretability and accuracy. Variables were further ranked by their frequency and order of selection across cross-validation cycles, refining the regression models.

**Figure 1:**
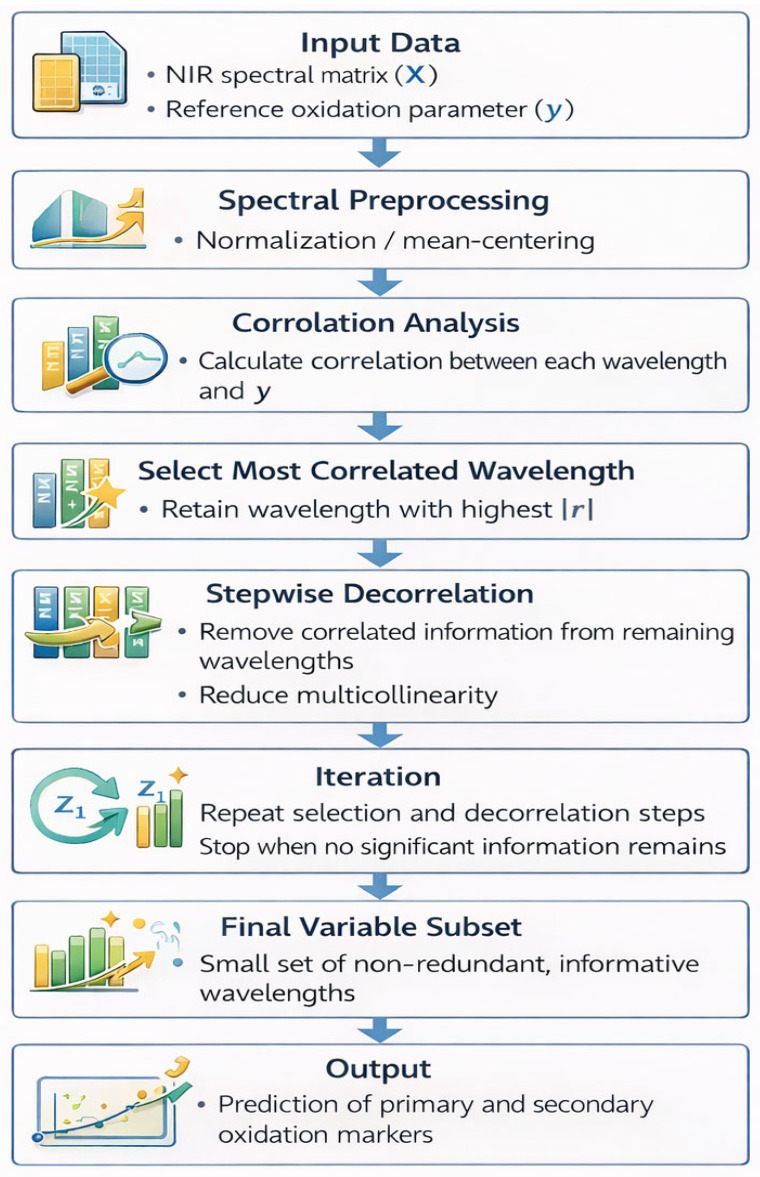
Flow chart illustrating the SELECT (Stepwise Decorrelation of Variables) algorithm applied to NIR spectral data. The process begins with spectral preprocessing and correlation analysis between each wavelength and the target response variable. The wavelength with the highest correlation is selected first, after which all remaining variables are decorrelated from the selected wavelength to remove redundant information. This stepwise selection–decorrelation procedure is repeated iteratively until a stopping criterion is reached. The final subset of orthogonal and informative wavelengths is then used to construct robust predictive models using ordinary least squares (OLS) regression.

This approach was used to optimize prediction of oxidation attributes in oil samples. SELECT was first applied to isolate the most relevant wavelengths, and full-spectrum OLS models were then built to quantify the primary and secondary oxidation markers [17,22]. Model performance was assessed using leave-one-out cross-validation (LOO), focusing on residual standard deviation, mean prediction error, mean absolute error (MAE), and the multiple correlation coefficient (R). The most effective model was chosen based on the lowest LOO prediction error. To ensure robust predictions and avoid overfitting, the number of predictors in the SELECT-OLS model was carefully optimized through validation procedures (Figure 1).

## 3. Results and Discussion

### 3.1. Primary and Secondary Oxidation Attributes

**Figure 2:**
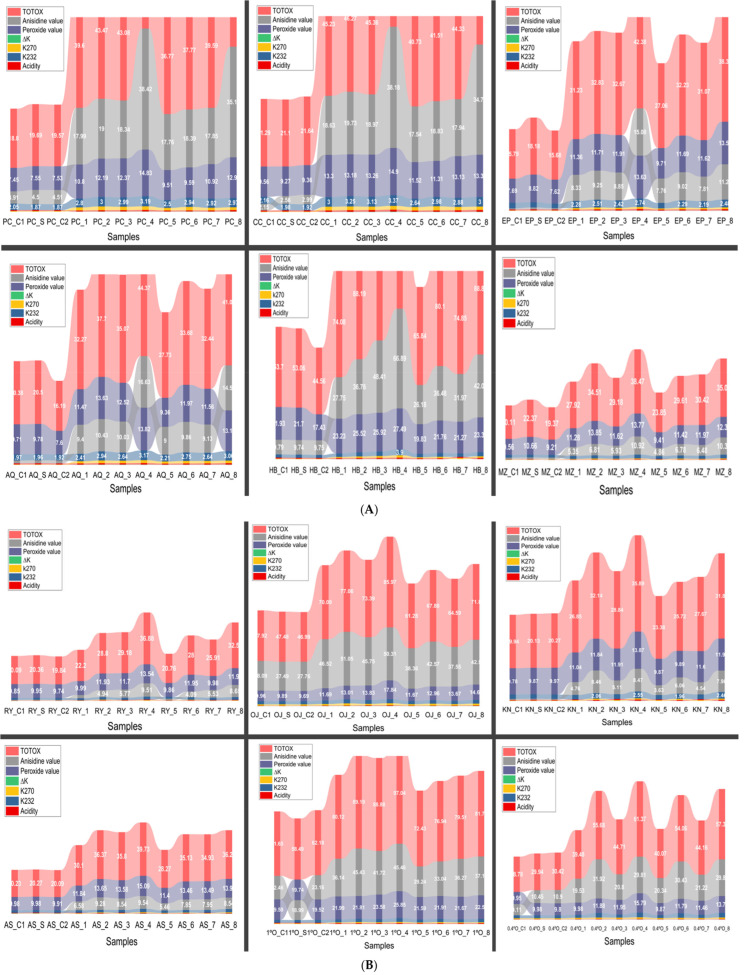

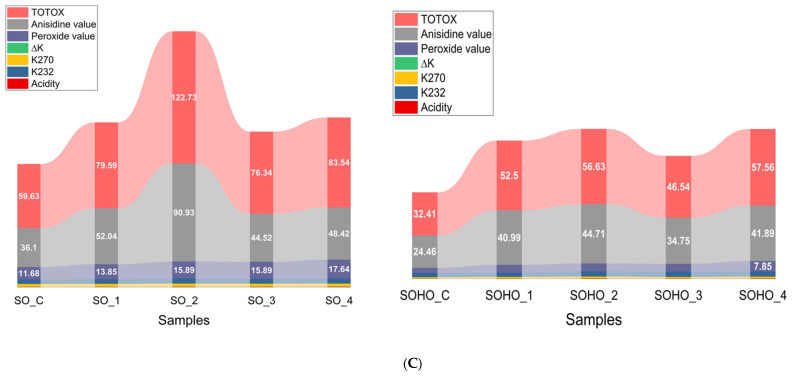
Changes in primary and secondary oxidation markers in various edible oils during deep frying. (**A**) Picual (PC), Cornicabra (CC), Empeltre (EP), Arbequina (AQ), Hojiblanca (HB), and Manzanilla (MZ). (**B**) Royuella (RY), Orujo (OJ), Koroneiki (KN), Arbosana (AS), Olive Oil 1% (1° O), and Olive Oil 0.4% (0.4° O). (**C**) Sunflower oil (SO) and high-oleic sunflower oil (SOHO). Oil sample treatment abbreviations are provided in Table S2.

The physicochemical properties of the oils varied substantially (Figure 2) depending on the treatment conditions, particularly deep frying temperature, duration, and the presence of polyphenol supplementation. Control samples (C1, C2, and S), representing non-fried oils, generally exhibited low values for acidity, K_232_, K_270_, ΔK, peroxide value, anisidine value, and TOTOX index, indicating minimal oxidative degradation. However, oils subjected to deep frying without supplementation (Experiments 1–4) exhibited a marked increase in all oxidative markers compared to the supplemented oil samples (Experiments 5–8), with the highest values consistently observed at 210 °C after 6 h of heating. These samples exhibited elevated acidity, increased conjugated diene and triene formation (K_232_ and K_270_), and substantial rises in both peroxide and anisidine values, leading to significantly higher TOTOX indices, which reflect overall oxidation. In contrast, olive oils supplemented with polyphenols (Experiments 5–8) demonstrated improved oxidative stability under the same frying conditions. While oxidative markers still increased compared to control levels, the rate and extent of degradation were notably reduced, confirming the protective effect of olive fruit extract. Among oil types, high-oleic sunflower oil (SOHO) and certain olive cultivars like EVOO Empeltre and EVOO Arbosana maintained better stability than regular sunflower oil (SO), which showed the highest levels of degradation. Overall, the data highlight that frying time and temperature are major drivers of oil oxidation, but polyphenol enrichment and the intrinsic properties of specific oils can substantially mitigate this deterioration.

Our findings, supported by previous studies [23,24], demonstrate the crucial role of phenolic compounds in preserving the oxidative stability and quality of olive oils. Virgin olive oils naturally rich in phenols showed lower acidity, reduced initial peroxide values, and greater resistance to oxidative degradation compared to refined oils, which often rely on added antioxidants. Under deep frying conditions, all oils exhibited increased oxidative markers—such as acidity, K_232_, K_270_, peroxide and anisidine values—especially at higher temperatures and longer durations. However, oils supplemented with olive-derived polyphenols, particularly extracts rich in hydroxytyrosol and oleuropein aglycone, showed significantly improved stability. These phenolic compounds, more resistant to thermal breakdown than α-tocopherol, effectively reduced oxidation rates and maintained better quality indices, even during prolonged heat exposure. Enzymatic hydrolysates from olive leaves emerged as particularly potent antioxidants, enhancing the stability of both refined and pomace oils under accelerated storage and frying. Among the oil types tested, high-phenol extra virgin olive oils and high-oleic sunflower oil outperformed regular sunflower oil in oxidative resistance. Overall, the combination of intrinsic oil composition and phenolic enrichment significantly mitigates thermal oxidation, emphasizing the importance of phenol content and antioxidant strategy in extending oil shelf life and functionality.

Moreover, all oxidation indices show strong positive correlations (r > 0.84). TOTOX is most strongly correlated with anisidine value (r = 0.981) and peroxide value (r = 0.967). Strong correlations between K_232_, K_270_, ΔK, and peroxide value in EVOOs confirm the progression from primary to secondary oxidation during deep frying (Table S1).

### 3.2. Chemometrics Models for Acidity Quantification

To quantify the acidity levels of various olive oil and sunflower oil types using NIR spectroscopy, predictive models were developed by integrating the SELECT-OLS algorithm with targeted variable selection procedures. The current analysis centered on acidity as a key indicator of oil degradation. Table 1A presents the most informative wavelengths selected from the original 700 variables recorded in the NIR system. The model identified a subset of 23 latent wavelengths, with 1792 nm ranked as the most influential predictor based on its weight and regression coefficient. This wavelength is particularly sensitive to chemical changes associated with acidity, aligning with the O-H and C-H overtone and combination bands involved in lipid oxidation and hydrolysis.

The NIR region between 1700 and 2000 nm is especially relevant for detecting subtle changes in oil quality. Absorption patterns in this region reflect vibrational overtones associated with hydroperoxides (primary oxidation products) and free fatty acids, both of which influence acidity.

The wavelength identified in our study, around 1792 nm, corresponds closely to the 1760 nm region reported in previous research, which is associated with the first overtone of C–H stretching vibrations in –CH_2_, –CH_3_, and =CH_2_ groups of fatty acid chains. This region is sensitive to primary oxidation products and reflects changes in lipid hydrocarbon chains during oxidative degradation. The slight shift to 1792 nm can be attributed to differences in oil type, thermal treatment, and instrument calibration, but it similarly captures oxidation dynamics and hydrolysis processes, confirming that our NIR-SELECT-OLS models are effectively targeting chemical changes relevant to primary oxidation [13].

Oils subjected to thermal treatment, particularly deep frying at elevated temperatures and prolonged durations, exhibited decreased absorbance at selected wavelengths such as 1792 nm, correlating with increased acidity. For instance, control samples of extra virgin olive oils (e.g., Picual_Control 1, Hojiblanca_Control 2) showed higher absorbance and lower acidity values, while heavily fried samples (e.g., Olive 1°_Exp 4 or Hojiblanca_Exp 4) demonstrated the opposite trend, indicating advanced lipid degradation.

The performance of the developed SELECT-OLS model is detailed in Table 1B. The model demonstrates excellent predictive capability, with a multiple correlation coefficient (R) of 0.96, confirming that over 92% of the variability in acidity is explained by the selected spectral data. The model also yielded a low standard deviation of the error (0.04) and a MAE of 0.03, underscoring its accuracy. Leave-one-out (LOO) cross-validation further validated model robustness, with an explained variance of 89.21% and a residual standard deviation of 0.05.

In conclusion, the findings confirm that NIR spectroscopy, when combined with a chemometrically optimized model, provides a rapid, non-destructive, and reliable approach for predicting acidity levels in edible oils. This method also proves valuable for quality control and process monitoring in the olive and edible oils sector, as previously reported [25,26]. The selected wavelengths—especially in the 1700–1800 nm range—capture essential information related to oxidation and hydrolysis processes, making this approach well-suited for quality monitoring across different oil varieties, processing treatments, and storage conditions.

### 3.3. Chemometrics Models for K_232_ Quantification

To monitor the oxidative status and conjugated dienes (K_232_ index) in various olive and sunflower oil samples, a SELECT-OLS regression model was developed using NIR spectral data, with a particular focus on the 1392 nm region. This wavelength emerged as a key predictor in the optimal model, reflecting its sensitivity to C-H combination overtones associated with unsaturated fatty acid degradation and lipid oxidation processes.

The detailed chemometric variable selection procedure (Table 2A) reveals that the 1392 nm wavelength (predictor index 147) was the first and most heavily weighted variable selected (weight 0.73), with a strong negative correlation coefficient (−109.72).

The wavelength at 1392 nm is primarily associated with the –CH_3_ functional group, reflecting a combination of C–H stretching and C–H deformation vibrations (2C–H stretching + C–H deformation). This region is sensitive to variations in fatty acid composition, making it valuable for differentiating oils and characterizing fatty acid profiles [27,28]. In our study, this wavelength was identified by the SELECT algorithm as one of the most informative for predicting oxidation-related parameters, suggesting that subtle changes in methyl group environments—caused by oxidation or thermal stress—can contribute to the discrimination of oil types and fatty acid composition. These results are consistent with previous studies reporting the usefulness of the 1400 nm region in oil differentiation and fatty acid characterization.

This indicates that absorbance at this wavelength decreases as K_232_ values increase, consistent with the oxidation-induced degradation of unsaturated bonds in fatty acids. Subsequent selected wavelengths span from 1330 nm to 2430 nm, each contributing to capturing subtle spectral variations associated with oil composition and oxidative changes.

For example, sunflower oil samples (SO and SOHO) exhibited the highest K_232_ values (up to 4.69), indicative of severe primary oxidation, and correspondingly showed the lowest absorbance values (~0.3840–0.3939) at 1392 nm. Conversely, control samples such as EVOO Manzanilla (MZ_C1) and EVOO Royuela (RY_C2) oils had low K_232_ indices (1.35–1.65) and higher absorbance values (~0.4082–0.4108), demonstrating minimal oxidative degradation. These opposing trends validate the diagnostic relevance of the 1392 nm band as a robust marker of oxidative status.

Statistically, the developed SELECT-OLS model demonstrated excellent predictive performance (Table 2B). Key metrics include a high multiple correlation coefficient (R = 0.97), low mean absolute error (MAE = 0.14), and a residual variance below 8% in leave-one-out cross-validation, indicating strong generalization ability. The explained variance of 92.17% further confirms that the model effectively captures the spectral-chemical relationship underlying K_232_ variations across diverse oil types and frying conditions.

Overall, the integration of chemometric variable selection and regression modeling confirms that NIR spectroscopy, particularly absorption around 1392 nm, offers a rapid, non-destructive, and reliable approach for estimating conjugated diene content and assessing oxidative stability in oils. This capability is critical for quality control during processing and storage, allowing for timely detection of oxidative damage and maintenance of oil quality.

### 3.4. Chemometrics Models for K_270_ Index, ∆K, and PV Quantification

To assess the oxidative status of the oil samples further, the absorbance at 270 nm (K_270_ index), which reflects secondary oxidation products such as conjugated trienes, was also analyzed alongside the NIR spectral region around 2114 nm. This wavelength was chosen due to its sensitivity to chemical changes related to oil degradation and oxidation.

The dataset indicates that K_270_ values varied markedly across oil types and treatment conditions, with higher K_270_ values reflecting greater secondary oxidation and lipid degradation. For example, sunflower oil samples (SO series) exhibited the highest K_270_ values, ranging from 2.47 to 2.72, consistent with substantial formation of secondary oxidation products after thermal stress. These samples also showed elevated absorbance at 2114 nm (~1.126), corresponding to the C–H stretching and C=C stretching vibrations of HC=CH– groups, which are indicative of aldehyde and ketone formation arising from lipid degradation [13,27,28].

In contrast, control and less oxidized olive oil samples, such as Picual (PC) and Cornicabra (CC), displayed significantly lower K_270_ values (~0.13–0.20) along with lower absorbance at 2114 nm (~1.079–1.088). This reflects their preserved quality and minimal oxidative damage, demonstrating that the NIR signal at 2114 nm effectively tracks the accumulation of secondary oxidation products and can differentiate oils according to their oxidative status (Table 3A).

Intermediate values were observed for other oil types and treatments, such as Arbequina (AQ), Hojiblanca (HB), Manzanilla (MZ), and high-oleic sunflower oils (SOHO), highlighting a gradient of oxidative degradation linked with their deep frying conditions.

This correlation between K_270_ and NIR absorbance at 2114 nm supports the use of NIR spectroscopy as a non-destructive technique to monitor oil quality, specifically tracking the formation of secondary oxidation products. Absorbance changes in the 2114 nm region can thus serve as a proxy for oxidative stability, complementing traditional UV indices such as K_270_.

Overall, these findings (Table 3B) emphasize the complementary roles of spectral analysis and chemical indices like K_270_ for comprehensive quality assessment. This approach allows rapid, real-time monitoring of oxidative changes in various oils during processing, storage, and thermal exposure, aiding in better quality control and shelf-life prediction.

The chemometric model for ∆K developed via SELECT-OLS from column auto-scaled NIR spectra (Table 4) demonstrates excellent predictive capacity, with a correlation coefficient R = 0.97 and an explained variance of ~92.6%. Selected wavelengths near 2118 nm, corresponding to overtone absorptions of CH and OH groups, suggest the model effectively captures chemical changes linked to oxidation and antioxidant depletion.

In addition to Delta K (∆K), which reflects secondary oxidation products, the PV was measured across a wide range of olive oil cultivars and treatment conditions to assess primary oxidation products—critical indicators of oil quality and shelf life. The PV data (Table 5) reveal significant variation dependent on cultivar type and thermal treatment.

High-oleic sunflower oil (SOHO) consistently exhibits low PVs (~4–8 meq O_2_/kg), indicative of its inherent oxidative stability. Conversely, oils subjected to prolonged heating or originating from less stable cultivars—such as Hojiblanca (HB) and refined olive oils (1ºO)—show elevated PVs reaching ~17–27 and ~19–26 meq O_2_/kg, respectively. Control samples (C1, S, C2) typically maintain lower PVs (~7–10 meq O_2_/kg), demonstrating the protective effect of polyphenol supplementation, while samples exposed to intensive thermal stress (e.g., PC_4, HB_4, 1ºO_4) show PVs exceeding 20 meq O_2_/kg, reflecting ongoing lipid peroxidation.

Oxidation in olive oil leads to the formation of compounds that can be identified using spectroscopic methods, especially infrared [29], as also reported by Guzmán et al. [30] and Tena et al. [31]. These compounds are closely linked to the development of rancid off-flavors [32].

Correlation analysis between PV and ∆K values highlights their complementary roles as oxidation markers. Samples with elevated peroxide content generally exhibit increased ∆K values, confirming a progression from primary to secondary oxidation products. For example, Hojiblanca oils (HB) with PVs above 20 meq O_2_/kg correspond to ∆K values up to 0.32, while high-oleic sunflower oils (SOHO) maintain both low PV and ∆K values, indicating superior oxidative stability.

Together, this integrated approach—combining rapid, non-destructive NIR spectroscopy with robust chemometric modeling—provides a powerful tool for monitoring oil oxidation under various conditions, including frying. It facilitates the evaluation of polyphenol supplementation efficacy and enhances quality control by offering comprehensive insight into the oxidative status of oils, from early peroxide formation to secondary conjugated dienes. This dual-marker strategy enables improved shelf-life prediction and better-informed decisions in oil deep frying processing.

### 3.5. Chemometrics Models for Anisidine Value (AnV) and TOTOX Index Quantification

The AnV and TOTOX index serve as critical complementary parameters for assessing the oxidative status of oils, particularly focusing on secondary oxidation products. While AnV quantifies aldehydes, mainly 2-alkenals formed during lipid oxidation, the TOTOX index integrates primary (peroxide value) and secondary (AnV) oxidation markers, providing a holistic measure of oil deterioration.

The data (Figure 2) reveal that control samples (e.g., PC_C1, CC_C1) exhibit low AnV values (approximately 2–4), consistent with minimal secondary oxidation and good oil quality. Polyphenol-supplemented samples (PC_S, CC_S) maintain similarly low AnV values, supporting the protective antioxidant effect of supplementation.

In contrast, oils subjected to prolonged or intense thermal stress show marked increases in AnV. For instance, samples such as PC_4, CC_4, and HB_4 register notably high AnV values, reaching 35–67, signaling substantial aldehyde formation and advanced oxidation. This is particularly evident in cultivars known for lower oxidative stability, like Hojiblanca (HB), where AnV reaches values above 60 under extended deep frying.

The TOTOX index parallels AnV trends closely, with values nearly doubling in stressed samples compared to controls. For example, HB_4 exhibits a TOTOX value of 121.66, reflecting cumulative primary and secondary oxidation. This high TOTOX corresponds well with the elevated peroxide and Delta K values previously discussed, indicating severe oxidative degradation.

Lower AnV and TOTOX values in EVOO Empeltre (EP) and other more stable cultivars highlight inherent resistance to oxidation, which is further enhanced by polyphenol supplementation. Moreover, high-oleic sunflower oils (SOHO) and similar oils display moderate AnV and TOTOX values, reflecting their better oxidative stability despite some thermal exposure.

Overall, AnV and TOTOX indices underscore the progression of lipid oxidation from primary peroxides to reactive aldehydes. Their combined evaluation is crucial for a comprehensive understanding of oil quality, confirming that polyphenol supplementation effectively mitigates oxidative damage under frying conditions. These parameters, alongside NIR-based ∆K and PV measurements, enable robust monitoring of oil stability and shelf life.

The chemometric analysis detailed in Table 6 and Table 7 demonstrates the efficacy of the SELECT variable selection combined with ordinary least squares regression for predicting key oxidation parameters—AnV and TOTOX—across different oil types, including extra virgin olive oils, refined and virgin olive oils, sunflower oil, and high-oleic sunflower oil.

The variable selection procedure in both models highlights specific NIR wavelengths strongly correlated with the oxidation indices. For AnV (Table 6), the most relevant wavelengths cluster primarily between 1150 and 2450 nm, with the highest weighted predictors located around 1392 nm (predictor index 147), 1970 nm (index 436), and 2362 nm (index 632). These wavelengths likely correspond to molecular vibrations related to aldehydes and secondary oxidation products, which directly impact the AnV.

In addition, the absorption band at 1392 nm arises from the combination of C–H stretching and deformation vibrations of –CH_2_– and –CH_3_ groups in linear alkane chains, reflecting the methyl and methylene content of fatty acids. The bands observed between 2120 and 2176 nm can be attributed to combination bands of C–H stretching vibrations from –HC=CH– (cis) groups of unsaturated fatty acids, as well as the combined absorption of C=O stretching vibrations. These regions are therefore sensitive to both the degree of unsaturation and the presence of carbonyl-containing oxidation products, enabling NIR spectroscopy to detect structural changes associated with lipid oxidation and thermal degradation [33].

Similarly, the TOTOX model (Table 7) selected wavelengths also concentrate within a similar spectral region, emphasizing bands near 1394 nm (index 148), 1970 nm (index 436), and 2254–2404 nm (indices 578, 633, 653). The selected predictors indicate the combined sensitivity of TOTOX to both primary (peroxide value) and secondary oxidation products (anisidine value), consistent with the known chemical nature of the TOTOX index.

The NIR region between 2100 and 2200 nm is associated with combination bands of C–H stretching in –HC=CH– (cis) groups of unsaturated fatty acids and C=O stretching vibrations of carbonyl compounds. Variations in absorbance within this region reflect differences in free fatty acid content, the degree of unsaturation, formation of trans-fatty acids, and the extent of lipid oxidation. In our study, oils subjected to thermal stress exhibited higher absorbance in this region, consistent with increased oxidation, hydrolysis, and structural alterations of unsaturated lipids, whereas less oxidized oils showed lower absorbance, indicating preserved chemical integrity. This demonstrates that the 2100–2200 nm spectral range is particularly sensitive to both compositional and oxidative changes, making it a valuable marker for monitoring oil quality and degradation under frying or storage conditions [34].

The weighting and correlation coefficients vary in magnitude and sign, reflecting the complex interplay of overlapping spectral features that influence the oxidation parameters. Notably, several wavelengths exhibit very high correlation coefficients, suggesting that these are critical for capturing the variation in AnV and TOTOX values.

Moreover, both models achieved strong performance metrics indicative of their robustness and reliability for practical application:

AnV model (Table 6) demonstrated excellent predictive ability with a multiple correlation coefficient (R) of 0.96 and a relatively low standard deviation of error (5.25). The Leave-One-Out (LOO) cross-validation results further confirmed model stability, with an explained variance of 86.5% and a modest MAE of 3.34.

TOTOX model (Table 7) also performed well, albeit with slightly lower accuracy, reflected in an R value of 0.90 and a higher error standard deviation (10.72). The LOO explained variance was 72.6%, demonstrating good but comparatively reduced model robustness for the more complex TOTOX parameter. The MAE of 7.37 suggests the model’s predictions are within acceptable limits for many practical quality control scenarios.

Higher complexity and larger error margin in the TOTOX model are expected, as TOTOX is a composite index incorporating both peroxide and anisidine values, inherently introducing more variability.

Furthermore, the detailed wavelength selection offers insight into the specific spectral regions most informative for oxidation-related compounds, supporting future refinement and development of tailored NIR sensor technologies.

Indeed, the traditional methods for assessing olive oil quality rely on chemical lab tests, which are costly, time-consuming, and generate waste [35]. Near-infrared spectroscopy (NIRS) has emerged as a promising alternative for rapid, on-site measurement of key quality parameters—free acidity, peroxide value, K_232_, and K_270_—with high accuracy [5]. Quality assessment models often use multivariate calibration techniques such as multiple linear regression, principal component regression, and most commonly, partial least squares (PLS) regression [36,37]. A recent advancement involves combining chemometric variable selection with NIRS, using targeted methods like SELECT-OLS to identify the most informative wavelengths [13,14,15]. This enhances model precision and computational efficiency [38].

The current results reveal that the targeted NIRS approach is especially valuable for industry, enabling fast, non-destructive quality control at olive oil or other edible oils. These systems can continuously update and improve their predictive models by incorporating new samples from different harvests, regions, and olive varieties. This adaptability ensures robust, precise quality control despite natural variability in oil samples and processing conditions.

The SELECT-OLS algorithm consistently achieves high predictive accuracy for various olive oil quality parameters. For acidity, it reported excellent metrics with a correlation coefficient (R) of 0.96, explaining over 92% of acidity variability through selected spectral variables. Similarly, the K_232_ parameter showed strong prediction performance with R = 0.97, 92.17% explained variance, and a low MAE of 0.14.

SELECT-OLS also demonstrated robustness across oxidation parameters: ΔK had R = 0.97 and ~92.6% explained variance, anisidine value showed R = 0.96 and MAE = 3.34, and the complex TOTOX index achieved R = 0.90 with MAE = 7.37.

A key advantage is its computational efficiency, selecting 23–30 optimal wavelengths from an initial 700 variables. This reduces complexity while maintaining or improving model accuracy compared to more complex methods, facilitating development of simplified, cost-effective NIR instruments for edible oil quality.

Leave-one-out cross-validation confirmed the models’ robustness, with explained variance from 86.5% to 92.6% across oxidation parameters, supporting their reliability on independent samples—a crucial factor for industrial quality control [38]. The method’s versatility extends to various oil types, including extra virgin olive oils, refined oils, and sunflower oils under diverse processing conditions [39].

By integrating rapid, non-destructive NIR spectroscopy with robust chemometric modeling, SELECT-OLS offers a powerful tool for real-time monitoring of oil oxidation during thermal processing and storage. Its high correlation and low errors enhance industrial quality control workflows, providing comprehensive insights into oil oxidative status from early peroxide formation to secondary conjugated dienes [40].

## 4. Conclusions

The integration of NIR spectroscopy with the SELECT chemometric algorithm and OLS regression proved highly effective for the rapid, non-destructive quantification of key quality and oxidation parameters in olive and sunflower oils. Out of the 700 measured wavelengths, only a subset was required for optimal prediction performance. Targeted variable selection revealed that 23 wavelengths were most relevant for acidity, 27 for K_232_ (conjugated dienes), 27 for K_270_ (conjugated trienes), 16 for ΔK, 30 for peroxide value (PV), 30 for AnV, and 22 for the TOTOX index. Specific wavelengths around 1792 nm for acidity and 1392 nm for K_232_ were identified as particularly informative, as they are closely linked to chemical changes resulting from lipid oxidation and hydrolysis. The predictive models exhibited strong performance metrics, with high correlation coefficients (R > 0.90) and explained variances exceeding 85% for acidity, primary and secondary oxidation products (PV, K_232_, K_270_, ΔK), and composite indices (AnV, TOTOX). These results confirm that the SELECT-OLS approach can reliably capture the spectral signatures associated with different stages of oil oxidation across various oil types, treatments, and deep frying conditions. Furthermore, the study demonstrated the complementary value of combining multiple oxidation markers and spectral features for comprehensive oil quality assessment. The ability to monitor oxidative degradation in real-time supports improved quality control during deep frying, as well as the evaluation of antioxidant supplementation effects. Overall, this work establishes NIR spectroscopy coupled with chemometric modeling as a powerful tool for ensuring edible oil quality, enhancing shelf-life prediction, and guiding better management practices in the food industry.

## Figures and Tables

**Table 1:** Chemometric summary of the variable selection and decorrelation process performed using the SELECT algorithm for the optimal OLS regression model based on column auto-scaled NIR spectra. The model was developed to predict acidity in extra virgin olive oils, refined and virgin olive oils, sunflower oil, and high-oleic sunflower oil. (**A**) Details of the variable selection process. (**B**) Statistical performance of the resulting regression model.

(A) SELECT-OLS Chemometric Modeling				
Order of Selection	Predictor Index	Wavelength (nm)	Weight	Coefficient
1	347	1792	0.36	−25.88
2	247	1592	0.26	27.00
3	129	1356	0.55	−109.59
4	509	2116	0.37	−7.21
5	44	1186	0.18	−36.34
6	318	1734	0.10	4.89
7	72	1242	0.17	−108.59
8	45	1188	0.15	230.90
9	133	1364	0.12	−98.56
10	436	1970	0.16	15.64
11	265	1628	0.17	198.39
12	339	1776	0.12	27.86
13	377	1852	0.11	−61.27
14	2	1102	0.10	81.84
15	305	1708	0.12	−14.97
16	307	1712	0.13	45.22
17	306	1710	0.11	25.26
18	563	2224	0.10	14.67
19	153	1404	0.09	−62.17
20	667	2432	0.09	−3.77
21	555	2208	0.09	−48.99
22	308	1714	0.08	−18.86
23	237	1572	0.06	−175.87
Intercept	0.39			
**(B) Statistical Characteristics**				
Standard Deviation of the Error		0.04		
Mean Absolute Error (MAE)		0.03		
Multiple Correlation Coefficient (R)		0.96		
Leave-One-Out Residual Variance (%)		10.79		
Leave-One-Out Residual Standard Deviation		0.05		
Leave-One-Out Explained Variance (%)		89.21		
Leave-One-Out Mean Prediction Error		0.04		

**Table 2:** Chemometric summary of the variable selection and decorrelation process performed using the SELECT algorithm for the optimal OLS regression model based on column auto-scaled NIR spectra. The model was developed to predict K_232_ values in extra virgin olive oils, refined and virgin olive oils, sunflower oil, and high-oleic sunflower oil. (**A**) Details of the variable selection process. (**B**) Statistical performance of the resulting regression model.

(A) SELECT-OLS Chemometric Modeling				
Order of Selection	Predictor Index	Wavelength (nm)	Weight	Coefficient
1	147	1392	0.73	−109.72
2	186	1470	0.41	140.07
3	662	2422	0.15	−23.88
4	378	1854	0.11	74.87
5	116	1330	0.13	106.25
6	158	1414	0.16	140.69
7	338	1774	0.17	−56.95
8	182	1462	0.11	−709.78
9	337	1772	0.10	169.81
10	634	2366	0.10	−28.80
11	578	2254	0.11	46.75
12	648	2394	0.09	−36.00
13	142	1382	0.08	−261.38
14	323	1744	0.08	−63.92
15	305	1708	0.08	−23.14
16	309	1716	0.08	110.93
17	348	1794	0.08	−214.07
18	666	2430	0.10	−46.45
19	292	1682	0.07	54.50
20	521	2140	0.08	−39.70
21	134	1366	0.07	−1023.57
22	573	2244	0.06	66.97
23	594	2286	0.06	−32.91
24	605	2308	0.08	88.33
25	386	1870	0.06	306.32
26	387	1872	0.06	−708.12
27	127	1352	0.06	1112.35
Intercept	2.55			
**(B) Statistical Characteristics**				
Standard Deviation of the Error		0.19		
Mean Absolute Error (MAE)		0.14		
Multiple Correlation Coefficient (R)		0.97		
Leave-One-Out Residual Variance (%)		7.83%		
Leave-One-Out Residual Standard Deviation		0.21		
Leave-One-Out Explained Variance (%)		92.17%		
Leave-One-Out Mean Prediction Error		0.17		

**Table 3:** Chemometric summary of the variable selection and decorrelation process performed using the SELECT algorithm for the optimal OLS regression model based on column auto-scaled NIR spectra. The model was developed to predict K_270_ values in extra virgin olive oils, refined and virgin olive oils, sunflower oil, and high-oleic sunflower oil. (**A**) Details of the variable selection process. (**B**) Statistical performance of the resulting regression model.

(A) SELECT-OLS Chemometric Modeling				
Order of Selection	Predictor Index	Wavelength (nm)	Weight	Coefficient
1	508	2114	0.78	42.57
2	403	1904	0.38	−6.07
3	695	2488	0.18	−16.03
4	39	1176	0.14	−57.43
5	396	1890	0.08	66.89
6	574	2246	0.07	15.55
7	648	2394	0.06	−12.37
8	578	2254	0.09	33.70
9	573	2244	0.07	−30.71
10	691	2480	0.06	27.11
11	472	2042	0.05	4.10
12	49	1196	0.17	−157.50
13	457	2012	0.11	183.41
14	455	2008	0.12	−666.19
15	467	2032	0.11	−469.33
16	521	2140	0.10	−43.14
17	503	2104	0.10	−228.13
18	292	1682	0.09	97.37
19	661	2420	0.08	21.50
20	632	2362	0.07	−20.71
21	476	2050	0.06	325.89
22	694	2486	0.06	−61.42
23	298	1694	0.06	−92.12
24	515	2128	0.06	58.74
25	491	2080	0.05	104.87
26	497	2092	0.05	−474.02
27	310	1718	0.05	21.33
Intercept	0.74			
**(B) Statistical Characteristics**				
Standard Deviation of the Error		0.13		
Mean Absolute Error (MAE)		0.09		
Multiple Correlation Coefficient (R)		0.98		
Leave-One-Out Residual Variance (%)		6.48%		
Leave-One-Out Residual Standard Deviation		0.15		
Leave-One-Out Explained Variance (%)		93.52%		
Leave-One-Out Mean Prediction Error		0.11		

**Table 4:** Chemometric summary of the variable selection and decorrelation process performed using the SELECT algorithm for the optimal OLS regression model based on column auto-scaled NIR spectra. The model was developed to predict ΔK values in extra virgin olive oils, refined and virgin olive oils, sunflower oil, and high-oleic sunflower oil. (**A**) Details of the variable selection procedure. (**B**) Statistical performance of the resulting regression model.

(A) SELECT-OLS Chemometric Modeling				
Order of Selection	Predictor Index	Wavelength (nm)	Weight	Coefficient
1	510	2118	0.84	5.22
2	416	1930	0.29	−2.08
3	414	1926	0.14	32.34
4	526	2150	0.13	−2.66
5	542	2182	0.16	10.96
6	516	2130	0.10	5.81
7	509	2116	0.08	34.28
8	569	2236	0.07	−3.43
9	326	1750	0.09	3.25
10	306	1710	0.08	−6.23
11	485	2068	0.10	6.89
12	507	2112	0.10	−71.66
13	413	1924	0.09	56.49
14	309	1716	0.09	11.25
15	670	2438	0.05	−1.23
16	304	1706	0.06	−6.86
Intercept	0.06951			
**(B) Statistical Characteristics**				
Standard Deviation of the Error			0.02	
Mean Absolute Error (MAE)			0.01	
Multiple Correlation Coefficient (R)			0.97	
Leave-One-Out Residual Variance (%)			7.44%	
Leave-One-Out Residual Standard Deviation			0.02	
Leave-One-Out Explained Variance (%)			92.56%	
Leave-One-Out Mean Prediction Error			0.02	

**Table 5:** Chemometric summary of the variable selection and decorrelation process performed using the SELECT algorithm for the optimal OLS regression model based on column auto-scaled NIR spectra. The model was developed to predict PVs in extra virgin olive oils, refined and virgin olive oils, sunflower oil, and high-oleic sunflower oil. (**A**) Details of the variable selection procedure. (**B**) Statistical performance of the resulting regression model.

(A) SELECT-OLS Chemometric Modeling				
Order of Selection	Predictor Index	Wavelength (nm)	Weight	Coefficient
1	139	1376	0.32	−800.99
2	209	1516	0.48	2534.35
3	223	1544	0.24	−4444.35
4	340	1778	0.23	392.88
5	41	1180	0.18	−761.72
6	181	1460	0.28	−1125.42
7	670	2438	0.22	−237.25
8	461	2020	0.18	836.18
9	186	1470	0.19	9138.76
10	349	1796	0.18	957.84
11	211	1520	0.12	−14,347.13
12	224	1546	0.12	10,930.75
13	326	1750	0.12	−417.96
14	625	2348	0.12	141.39
15	636	2370	0.10	−205.15
16	210	1518	0.09	−12,204.49
17	689	2476	0.09	−173.08
18	586	2270	0.10	−358.99
19	601	2300	0.09	−800.99
20	604	2306	0.10	2534.35
21	207	1512	0.09	−4444.35
22	311	1720	0.09	392.88
23	607	2312	0.08	−761.72
24	614	2326	0.08	−1125.42
25	603	2304	0.09	−237.25
26	691	2480	0.08	836.18
27	226	1550	0.07	9138.76
28	465	2028	0.09	957.84
29	357	1812	0.10	−14,347.13
30	367	1832	0.08	10,930.75
Intercept	13.05556			
**(B) Statistical Characteristics**				
Standard deviation of the error		1.73		
Mean absolute error (MAE)		1.22		
Multiple correlation coefficient (R)		0.94		
LEAVE-ONE-OUT residual variance		18.33%		
LEAVE-ONE-OUT residual standard deviation		1.97		
LEAVE-ONE-OUT explained variance		81.67%		
LEAVE-ONE-OUT mean prediction error		1.57		

**Table 6:** Chemometric summary of the variable selection and decorrelation process performed using the SELECT algorithm for the optimal OLS regression model based on column auto-scaled NIR spectra. The model was developed to predict AnV values in extra virgin olive oils, refined and virgin olive oils, sunflower oil, and high-oleic sunflower oil. (**A**) Details of the variable selection procedure. (**B**) Statistical performance of the resulting regression model.

(A) SELECT-OLS Chemometric Modeling				
Order of Selection	Predictor Index	Wavelength (nm)	Weight	Coefficient
1	147	1392	0.65	−2139.32
2	436	1970	0.49	3945.58
3	632	2362	0.14	−378.30
4	158	1414	0.12	901.97
5	378	1854	0.11	2232.23
6	120	1338	0.22	3987.53
7	678	2454	0.12	716.74
8	41	1180	0.10	−3330.91
9	143	1384	0.08	6173.80
10	500	2098	0.09	−1536.66
11	578	2254	0.08	651.67
12	246	1590	0.08	6199.57
13	231	1560	0.09	−25,831.90
14	306	1710	0.09	−525.99
15	307	1712	0.08	1516.80
16	265	1628	0.08	5421.99
17	305	1708	0.14	−3297.27
18	437	1972	0.08	−18,201.29
19	129	1356	0.08	−24,289.36
20	269	1636	0.07	17,533.14
21	303	1704	0.07	−1422.31
22	28	1154	0.09	10,124.97
23	333	1764	0.08	−2619.12
24	237	1572	0.07	−32,047.14
25	111	1320	0.08	−25,503.10
26	154	1406	0.06	−9077.43
27	72	1242	0.07	−14,594.19
28	73	1244	0.07	30,340.46
29	150	1398	0.07	23,577.69
30	252	1602	0.06	28,613.07
Intercept	18.47035			
**(B) Statistical Characteristics**				
Standard deviation of the error		5.25		
Mean absolute error (MAE)		3.34		
Multiple correlation coefficient (R)		0.96		
LEAVE-ONE-OUT residual variance		13.48%		
LEAVE-ONE-OUT residual standard deviation		6.17		
LEAVE-ONE-OUT explained variance		86.52%		
LEAVE-ONE-OUT mean prediction error		4.35		

**Table 7:** Chemometric summary of the variable selection and decorrelation process performed using the SELECT algorithm for the optimal OLS regression model based on column auto-scaled NIR spectra. The model was developed to predict TOTOX values in extra virgin olive oils, refined and virgin olive oils, sunflower oil, and high-oleic sunflower oil. (**A**) Details of the variable selection procedure. (**B**) Statistical performance of the resulting regression model.

(A) SELECT-OLS Chemometric Modeling				
Order of Selection	Predictor Index	Wavelength (nm)	Weight	Coefficient
1	148	1394	0.55	−2355.09
2	436	1970	0.55	6063.79
3	653	2404	0.14	−746.72
4	578	2254	0.12	1201.14
5	633	2364	0.15	−1190.38
6	378	1854	0.09	2575.18
7	249	1596	0.18	4790.37
8	41	1180	0.17	−8532.07
9	579	2256	0.09	−1499.60
10	584	2266	0.10	1030.04
11	597	2292	0.11	−2993.93
12	601	2300	0.08	3237.23
13	586	2270	0.09	−2698.39
14	606	2310	0.07	2828.94
15	604	2306	0.08	−4273.12
16	248	1594	0.06	−37,674.22
17	594	2286	0.05	−2453.66
18	591	2280	0.08	4671.52
19	610	2318	0.06	2308.80
20	593	2284	0.06	−2615.21
21	596	2290	0.06	3195.97
22	603	2304	0.06	3438.93
Intercept	44.56894			
**(B) Statistical Characteristics**				
Standard deviation of the error		10.72		
Mean absolute error (MAE)		7.37		
Multiple correlation coefficient (R)		0.90		
LEAVE-ONE-OUT residual variance		27.36%		
LEAVE-ONE-OUT residual standard deviation		11.99		
LEAVE-ONE-OUT explained variance		72.64%		
LEAVE-ONE-OUT mean prediction error		8.88		

## Data Availability

The original contributions presented in this study are included in the article/Supplementary Material. Further inquiries can be directed to the corresponding author.

## Supplementary Materials

The following supporting information can be downloaded at: https://www.mdpi.com/article/10.3390/foods15030557/s1, Figure S1. NIR spectra of various edible oils under frying conditions, including different extra-virgin olive oil (EVOO) cultivars; EVOO/VOO blends with refined olive oil (ROO); pomace olive oil (orujo); sunflower oil; and high-oleic sunflower oil. Spectra of fried olive oils, supplemented or unsupplemented with hydroxytyrosol (HTyr), are compared with those of non-fried olive oils, both supplemented and unsupplemented; Figure S2. % Residual variance of the variables decorrelated by SELECT approach obtained from auto-scaled NIR spectra, using the acidity (A), K_232_ (B), K_270_ (C), and delta K (D) as response variable; Figure S3. % Residual variance of the variables decorrelated by SELECT approach obtained from auto-scaled NIR spectra, using the PV (A), AnV (B), and TOTOX (C), as response variable; Table S1. Pearson correlation matrix between oxidation parameters in Picual and Cornicabra samples; Table S2. Mean values of primary and secondary oxidation markers—including acidity, K_232_, K_270_, ∆K, peroxide value, anisidine value, and TOTOX—in various edible oils during deep frying.

## References

[1] Mehany T., González-Sáiz J.M., Pizarro C. (2025). Enhanced Thermal Resilience of Olive Oils: Fatty Acid Dynamics with Polyphenols Supplementation. Foods.

[2] De Stefanis D., Costelli P. (2025). Extra Virgin Olive Oil (EVOO) Components: Interaction with Pro-Inflammatory Cytokines Focusing on Cancer and Skeletal Muscle Biology. Nutrients.

[3] Vidal Damasceno J., Garcez A., Anelo Alves A., da Mata I.R., Morelo Dal Bosco S., Garavaglia J. (2026). Effects of Daily Extra Virgin Olive Oil Consumption on Biomarkers of Inflammation and Oxidative Stress: A Systematic Review and Meta-Analysis. Crit. Rev. Food Sci. Nutr..

[4] Milena E., Maurizio M. (2025). Exploring the Cardiovascular Benefits of Extra Virgin Olive Oil: Insights into Mechanisms and Therapeutic Potential. Biomolecules.

[5] Abdellatif T.M.H. (2025). Stability of Extra Virgin Olive Oil and Other Edible Oils Under Deep-Frying and Storage Stress: A Multivariate Study of Instrumental and Sensory Data. Ph.D. Dissertation.

[6] del Río Celestino M., Font R. (2022). Using Vis-NIR Spectroscopy for Predicting Quality Compounds in Foods. Sensors.

[7] Arroyo-Cerezo A., Yang X., Jiménez-Carvelo A.M., Pellegrino M., Savino A.F., Berzaghi P. (2024). Assessment of Extra Virgin Olive Oil Quality by Miniaturized near Infrared Instruments in a Rapid and Non-Destructive Procedure. Food Chem..

[8] Zaukuu J.-L.Z., Adam M.N., Nkansah A.A., Mensah E.T. (2024). Detection and Quantification of Groundnut Oil Adulteration with Machine Learning Using a Comparative Approach with NIRS and UV–VIS. Sci. Rep..

[9] García Martín J.F. (2022). Potential of Near-Infrared Spectroscopy for the Determination of Olive Oil Quality. Sensors.

[10] Cayuela Sánchez J.A., Moreda W., García J.M. (2013). Rapid Determination of Olive Oil Oxidative Stability and Its Major Quality Parameters Using Vis/NIR Transmittance Spectroscopy. J. Agric. Food Chem..

[11] Michelucci U., Venturini F. (2024). Deep Learning Domain Adaptation to Understand Physico-Chemical Processes from Fluorescence Spectroscopy Small Datasets and Application to the Oxidation of Olive Oil. Sci. Rep..

[12] Garrido-Varo A., Sánchez M.-T., De la Haba M.-J., Torres I., Pérez-Marín D. (2017). Fast, Low-Cost and Non-Destructive Physico-Chemical Analysis of Virgin Olive Oils Using near-Infrared Reflectance Spectroscopy. Sensors.

[13] Mehany T., González-Sáiz J., Pizarro C. (2025). The Quality Prediction of Olive and Sunflower Oils Using NIR Spectroscopy and Chemometrics: A Sustainable Approach. Foods.

[14] Mehany T., González-Sáiz J.M., Pizarro C. (2025). Quantification of Phenolic Compounds in Olive Oils by Near-Infrared Spectroscopy and Multiple Regression: Effects of Cultivar, Hydroxytyrosol Supplementation, and Deep-Frying. Antioxidants.

[15] Mehany T., González-Sáiz J.M., Pizarro C. (2025). SELECT-OLS Calibration Models for Robust Fatty Acids Quantification in Various Vegetable Oils Using NIR Spectroscopy: A Unified Approach across Hydroxytyrosol Supplementation and Deep-Frying Conditions. LWT.

[16] Casale M., Oliveri P., Casolino C., Sinelli N., Zunin P., Armanino C., Forina M., Lanteri S. (2012). Characterisation of PDO Olive Oil Chianti Classico by Non-Selective (UV–Visible, NIR and MIR Spectroscopy) and Selective (Fatty Acid Composition) Analytical Techniques. Anal. Chim. Acta.

[17] Pizarro C., Esteban-Díez I., González-Sáiz J.-M., Forina M. (2007). Use of Near-Infrared Spectroscopy and Feature Selection Techniques for Predicting the Caffeine Content and Roasting Color in Roasted Coffees. J. Agric. Food Chem..

[18] Mehany T., González-Sáiz J.M., Martínez J., Pizarro C. (2024). Evaluation of Sensorial Markers in Deep-Fried Extra Virgin Olive Oils: First Report on the Role of Hydroxytyrosol and Its Derivatives. Foods.

[19] Commission Regulation (1991). Commission Regulation (EEC) No. 2568/91 of 11 July 1991 on the Characteristics of Olive Oil and Olive-Residue Oil and on the Relevant Methods of Analysis. Official Journal L 248.

[20] (1998). Official Methods and Recommended Practices of the American Oil Chemists Society.

[21] Aşkın B., Kaya Y. (2020). Effect of Deep Frying Process on the Quality of the Refined Oleic/Linoleic Sunflower Seed Oil and Olive Oil. J. Food Sci. Technol..

[22] Forina M., Lanteri S., Casale M., Cerrato Oliveros M.C. (2007). Stepwise Orthogonalization of Predictors in Classification and Regression Techniques: An “Old” Technique Revisited. Chemom. Intell. Lab. Syst..

[23] Satue M.T., Huang S., Frankel E.N. (1995). Effect of Natural Antioxidants in Virgin Olive Oil on Oxidative Stability of Refined, Bleached, and Deodorized Olive Oil. J. Am. Oil Chem. Soc..

[24] Bouaziz M., Feki I., Ayadi M., Jemai H., Sayadi S. (2010). Stability of Refined Olive Oil and Olive-pomace Oil Added by Phenolic Compounds from Olive Leaves. Eur. J. Lipid Sci. Technol..

[25] Bandiera A., Camerlingo A., Sanna N., Zazza C., Benelli A., Massantini R., Moscetti R. (2026). Comparing Deep and Classical Chemometrics: Can CNN Enhance the Accuracy of EVOO Adulteration Detection from Spectral Data?. Food Control.

[26] Luo W., Deng J., Jiang H., Chen Q. (2025). Comparison of Variable Optimization Algorithms for PLS Regression Models of Kerosene Content in Edible Oils. Spectrochim. Acta A Mol. Biomol. Spectrosc..

[27] Ding F., Sánchez-Villasclaras S., Pan L., Lan W., García-Martín J.F. (2024). Advances in Vibrational Spectroscopic Techniques for the Detection of Bio-Active Compounds in Virgin Olive Oils: A Comprehensive Review. Foods.

[28] Sinelli N., Casale M., Di Egidio V., Oliveri P., Bassi D., Tura D., Casiraghi E. (2010). Varietal Discrimination of Extra Virgin Olive Oils by Near and Mid Infrared Spectroscopy. Food Res. Int..

[29] Grigoletto I., Cevoli C., Koidis A., Gallina Toschi T., Valli E. (2025). Infrared Spectroscopy and Chemometrics for Predicting Commercial Categories of Virgin Olive Oils and Supporting the Panel Test. Food Res. Int..

[30] Guzmán E., Baeten V., Fernández Pierna J.A., García-Mesa J.A. (2011). Application of Low-Resolution Raman Spectroscopy for the Analysis of Oxidized Olive Oil. Food Control.

[31] Tena N., Aparicio R., García-González D.L. (2017). Virgin Olive Oil Stability Study by Mesh Cell-FTIR Spectroscopy. Talanta.

[32] Frankel E.N. (1983). Volatile Lipid Oxidation Products. Prog. Lipid Res..

[33] Vieira L.S., Assis C., de Queiroz M.E.L.R., Neves A.A., de Oliveira A.F. (2021). Building Robust Models for Identification of Adulteration in Olive Oil Using FT-NIR, PLS-DA and Variable Selection. Food Chem..

[34] Meng X., Yin C., Yuan L., Zhang Y., Ju Y., Xin K., Chen W., Lv K., Hu L. (2023). Rapid Detection of Adulteration of Olive Oil with Soybean Oil Combined with Chemometrics by Fourier Transform Infrared, Visible–Near-Infrared and Excitation–Emission Matrix Fluorescence Spectroscopy: A Comparative Study. Food Chem..

[35] Mehany T., González-Sáiz J.M., Pizarro C. (2025). Recent Advances in Spectroscopic Approaches for Assessing the Stability of Bioactive Compounds and Quality Indices of Olive Oil during Deep-Frying: Current Knowledge, Challenges, and Implications. Food Chem..

[36] Anto Win Shalini W., Rajalakshmi T., Vasanthadev Suryakala S. (2025). Enhancing the Diagnostic Evaluation of Thyroid Functionality Using Diffuse Reflectance Spectroscopy and Regression Models. J. Biophotonics.

[37] Karnachoriti M., Chatzipetrou M., Touloupakis E., Kontos A.G., Zergioti I. (2025). Raman Spectroscopy as a Tool for Real-Time Nutrient Monitoring in Bioreactor Cultivation of Microalgae. J. Raman Spectrosc..

[38] Bian X., Wang Y., Wang S., Johnson J.B., Sun H., Guo Y., Tan X. (2022). A Review of Advanced Methods for the Quantitative Analysis of Single Component Oil in Edible Oil Blends. Foods.

[39] Guirrou I., Kouighat M., Kettani R., Houmanat K., Kassimi C., El Harrak A., Nabloussi A. (2024). A Comprehensive Analysis of the Influence of Variety and Climate on Some Properties of Sunflower Oil. Acta Sci. Pol. Technol. Aliment..

[40] Osheter T., Campisi-Pinto S., Resende M., Linder C., Wiesman Z. (2022). 1H LF-NMR Self-Diffusion Measurements for Rapid Monitoring of an Edible Oil’s Food Quality with Respect to Its Oxidation Status. Molecules.

