# Supplementary material for: Rapid Monitoring and Quantification of Primary and Secondary Oxidative Markers in Edible Oils During Deep Frying Using Near-Infrared Spectroscopy and Chemometrics
Source: Foods. 2026 Feb 4;15(3):557. doi: 10.3390/foods15030557 (PMC12897350; doi:10.3390/foods15030557)
Supplement: Supplementary file 1 [file foods-15-00557-s001.zip › foods-4104195-supplementary.pdf]

Supplemenatry Data:

Article

Rapid Monitoring and Quantification of Primary and Secondary Oxidative Markers in Edible Oils During Deep Frying Using Near-Infrared Spectroscopy and Chemometrics

Taha Mehany, José M. González-Sáiz and Consuelo Pizarro *

Department of Chemistry, University of La Rioja, 26006 Logroño, Spain;
taha.abdellatif@unirioja.es (T.M.); josemaria.gonzalez@unirioja.es (J.M.G.-S.)
* Correspondence: consuelo.pizarro@unirioja.es

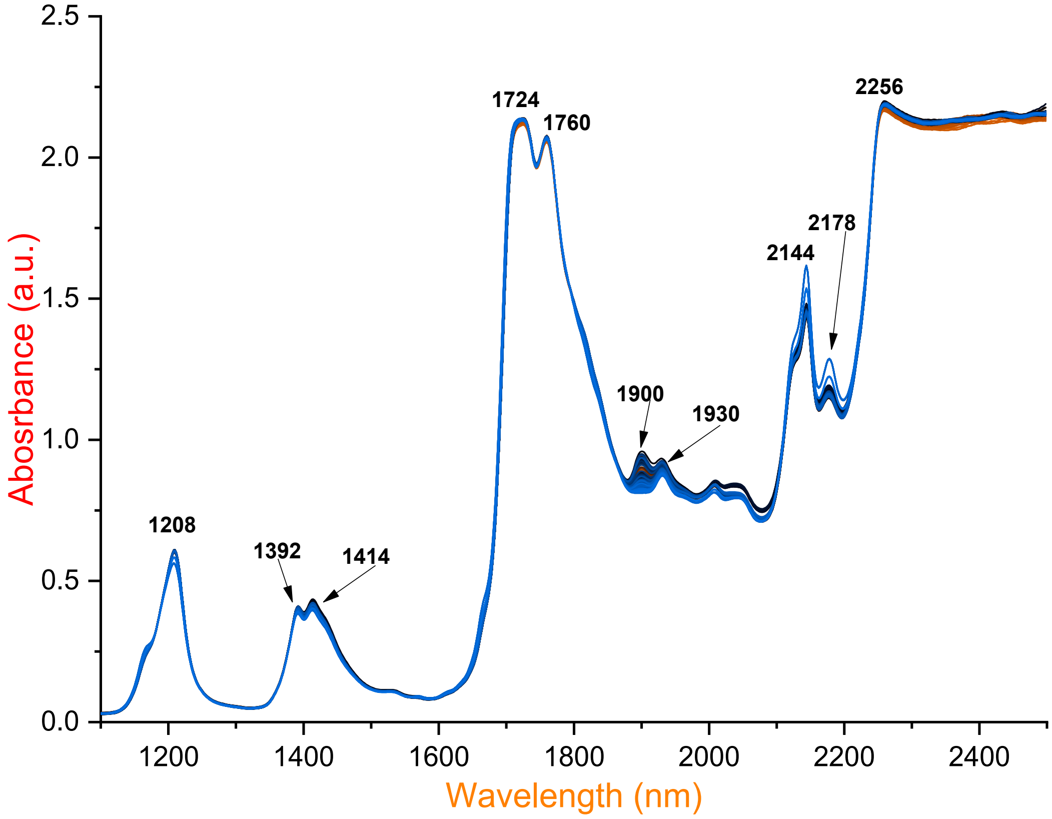

Figure S1. NIR spectra of various edible oils under frying conditions, including different extra-virgin olive oil (EVOO) cultivars; EVOO/VOO blends with refined olive oil (ROO); pomace olive oil (orujo); sunflower oil; and high-oleic sunflower oil. Spectra of fried olive oils, supplemented or unsupplemented with hydroxytyrosol (HTyr), are compared with those of non-fried olive oils, both supplemented and unsupplemented.

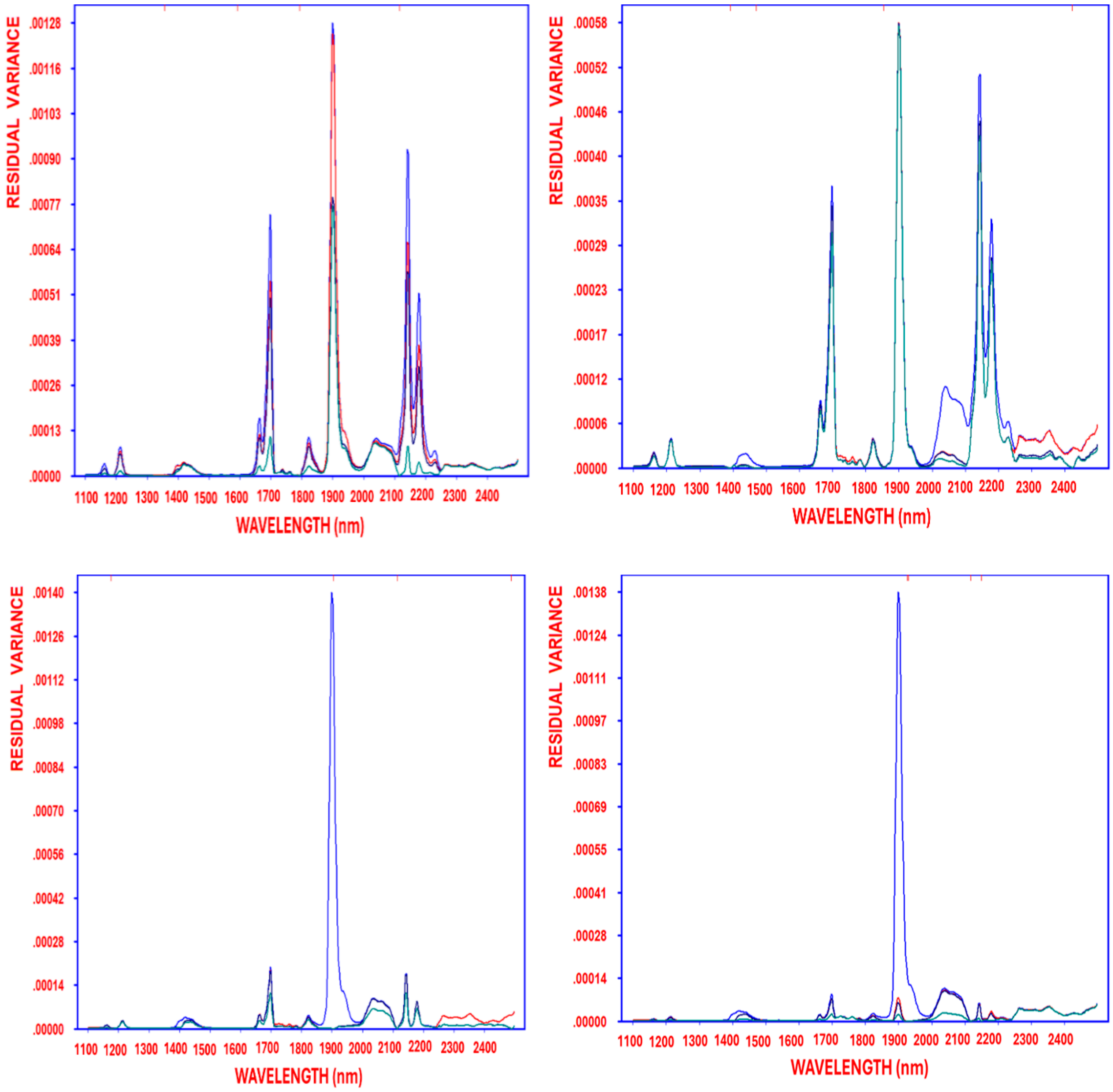

Figure S2. % Residual variance of the variables decorrelated by SELECT approach obtained from auto-scaled NIR spectra, using the acidity (A), K_{232} (B), K_{270} (C), and delta K (D) as response variable.

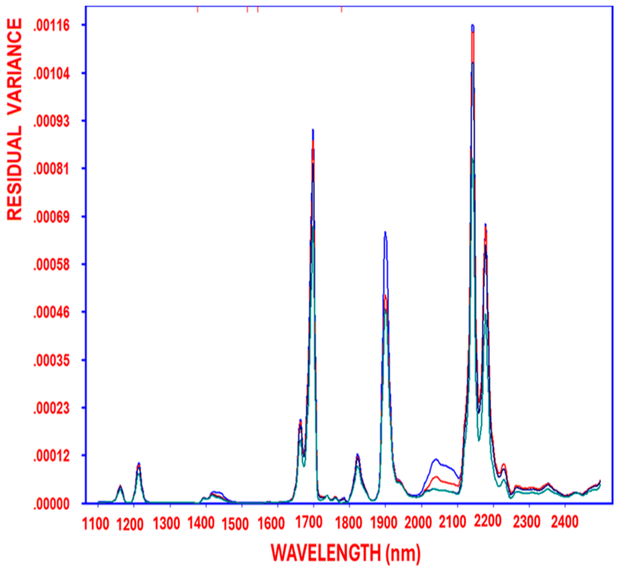

A

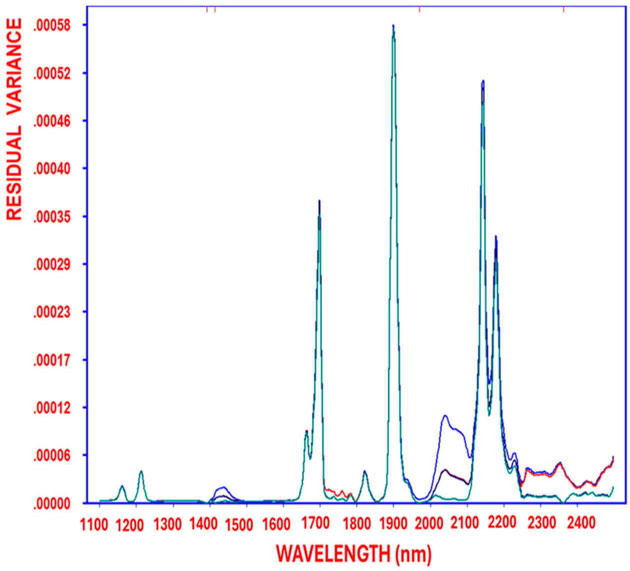

B

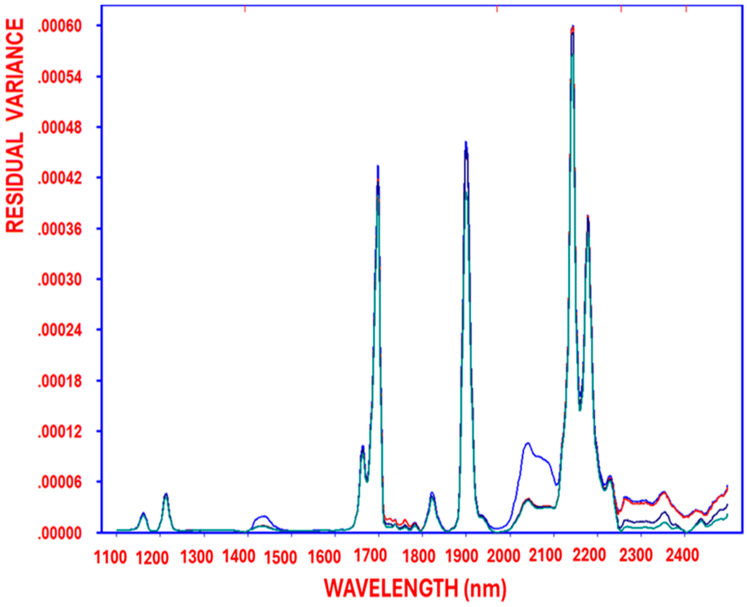

C

Figure S3. % Residual variance of the variables decorrelated by SELECT approach obtained from auto-scaled NIR spectra, using the PV (A), AnV (B), and TOTOX (C), as response variable.

Table S1. Pearson correlation matrix between oxidation parameters in Picual and Cornicabra samples.

Variable	Acidity	K_{232}	K_{270}	ΔK	Peroxide value	Anisidine value	TOTOX
Acidity	1.000	0.912	0.896	0.903	0.929	0.848	0.902
K_{232}	0.912	1.000	0.958	0.943	0.974	0.884	0.947
K_{270}	0.896	0.958	1.000	0.962	0.961	0.906	0.957
ΔK	0.903	0.943	0.962	1.000	0.950	0.896	0.949
Peroxide value	0.929	0.974	0.961	0.950	1.000	0.899	0.967
Anisidine value	0.848	0.884	0.906	0.896	0.899	1.000	0.981
TOTOX	0.902	0.947	0.957	0.949	0.967	0.981	1.000

Table S2. Mean values of primary and secondary oxidation markers—including acidity, K_{232}, K_{270}, ΔK, peroxide value, anisidine value, and TOTOX—in various edible oils during deep frying.

Samples	Acidity	K_{232}	K_{270}	ΔK	Peroxide value	Anisidine value	TOTOX
PC_C1	0.28	2.05	0.20	0.01	7.45	3.91	18.80
PC_S	0.33	1.87	0.20	0.00	7.55	4.50	19.69
PC_C2	0.28	1.87	0.20	0.01	7.53	4.51	19.57
PC_1	0.34	2.80	0.98	0.05	10.80	17.99	39.60
PC_2	0.39	3.00	1.20	0.07	12.19	19.00	43.47
PC_3	0.39	2.99	1.10	0.07	12.37	18.34	43.08
PC_4	0.48	3.19	1.15	0.07	14.83	38.42	67.99
PC_5	0.32	2.50	0.84	0.05	9.51	17.76	36.77
PC_6	0.33	2.94	1.12	0.07	9.59	18.39	37.77
PC_7	0.33	2.92	0.85	0.06	10.92	17.85	39.59
PC_8	0.44	2.97	0.89	0.06	12.91	35.11	60.94
CC_C1	0.33	2.16	0.13	0.01	9.56	2.15	21.29
CC_S	0.38	1.98	0.15	0.00	9.27	2.56	21.10
CC_C2	0.34	1.92	0.16	0.00	9.36	2.99	21.64
CC_1	0.44	3.00	1.18	0.06	13.30	18.63	45.23
CC_2	0.50	3.25	1.17	0.08	13.18	19.73	46.27
CC_3	0.55	3.13	1.06	0.08	13.26	18.97	45.36
CC_4	0.61	3.37	1.18	0.12	14.90	38.18	67.99
CC_5	0.38	2.64	1.12	0.07	11.52	17.54	40.73
CC_6	0.45	2.98	1.27	0.08	11.31	18.83	41.51
CC_7	0.50	2.88	1.05	0.06	13.13	17.94	44.33
CC_8	0.56	3.00	1.07	0.08	13.34	34.73	61.24
EP_C1	0.28	1.77	0.10	0.00	7.69	0.38	15.79
EP_S	0.33	1.67	0.11	0.01	8.82	0.59	18.18
EP_C2	0.28	1.44	0.10	0.00	7.62	0.45	15.68
EP_1	0.33	2.28	0.47	0.04	11.36	8.33	31.23
EP_2	0.38	2.51	0.59	0.05	11.71	9.25	32.83
EP_3	0.39	2.42	0.47	0.06	11.91	8.85	32.67
EP_4	0.50	2.74	0.63	0.06	13.63	15.08	42.38
EP_5	0.33	1.79	0.35	0.03	9.71	7.76	27.06
EP_6	0.37	2.29	0.54	0.05	11.69	9.02	32.23
EP_7	0.38	2.19	0.46	0.04	11.62	7.81	31.07
EP_8	0.45	2.48	0.55	0.06	13.55	11.27	38.38
AQ_C1	0.33	1.97	0.11	0.01	9.71	0.95	20.38
AQ_S	0.38	1.96	0.11	0.00	9.78	0.97	20.50
AQ_C2	0.33	1.92	0.11	0.00	7.60	0.99	16.19
AQ_1	0.39	2.41	0.71	0.06	11.47	9.40	32.27
AQ_2	0.45	2.94	0.88	0.07	13.63	10.43	37.70
AQ_3	0.43	2.64	0.82	0.07	12.52	10.03	35.07
AQ_4	0.49	3.17	0.92	0.07	13.82	16.63	44.37
AQ_5	0.34	2.21	0.69	0.05	9.36	9.00	27.73

AQ_6	0.38	2.75	0.83	0.06	11.97	9.86	33.68
AQ_7	0.39	2.64	0.81	0.05	11.56	9.13	32.44
AQ_8	0.44	3.06	0.85	0.07	13.18	14.56	41.03
HB_C1	0.60	2.16	0.28	0.10	21.93	9.79	53.70
HB_S	0.61	2.11	0.27	0.00	21.70	9.74	53.06
HB_C2	0.56	2.01	0.24	0.00	17.43	9.75	44.56
HB_1	0.62	2.41	0.70	0.10	23.23	27.75	74.08
HB_2	0.67	2.77	0.97	0.11	25.52	36.76	88.19
HB_3	0.71	3.00	1.23	0.12	25.92	48.41	99.84
HB_4	0.84	3.90	1.58	0.12	27.49	66.89	121.66
HB_5	0.56	2.31	0.68	0.01	19.83	26.18	65.84
HB_6	0.60	2.51	0.90	0.05	21.76	36.48	80.10
HB_7	0.71	2.30	0.70	0.06	21.27	31.97	74.85
HB_8	0.78	2.74	0.96	0.08	23.37	42.07	88.81
MZ_C1	0.17	1.65	0.16	0.00	9.56	0.97	20.11
MZ_S	0.23	1.62	0.18	0.00	10.66	1.03	22.37
MZ_C2	0.17	1.63	0.18	0.00	9.21	0.96	19.37
MZ_1	0.23	1.86	0.26	0.01	11.28	5.35	27.92
MZ_2	0.28	1.92	0.44	0.02	13.85	6.81	34.51
MZ_3	0.28	1.86	0.29	0.05	11.62	5.93	29.18
MZ_4	0.34	2.26	0.45	0.06	13.77	10.92	38.47
MZ_5	0.22	1.81	0.22	0.00	9.41	4.86	23.85
MZ_6	0.28	1.87	0.42	0.02	11.42	6.78	29.61
MZ_7	0.27	1.81	0.28	0.04	11.97	6.48	30.42
MZ_8	0.33	2.22	0.45	0.05	12.35	10.33	35.02
RY_C1	0.17	1.35	0.07	0.00	9.85	0.35	20.09
RY_S	0.22	1.36	0.06	0.01	9.95	0.42	20.36
RY_C2	0.17	1.35	0.07	0.00	9.74	0.36	19.84
RY_1	0.27	1.41	0.11	0.00	9.99	2.22	22.20
RY_2	0.33	1.79	0.37	0.03	11.93	4.94	28.80
RY_3	0.38	1.60	0.15	0.01	11.70	5.77	29.18
RY_4	0.39	2.06	0.38	0.04	13.54	9.51	36.88
RY_5	0.27	1.36	0.11	0.00	9.86	1.03	20.76
RY_6	0.33	1.76	0.33	0.03	11.95	4.09	28.00
RY_7	0.34	1.68	0.13	0.00	9.98	5.53	25.91
RY_8	0.34	1.92	0.27	0.03	11.94	8.65	32.54
OJ_C1	0.28	3.07	1.21	0.15	9.96	28.09	47.92
OJ_S	0.28	3.01	1.28	0.10	9.89	27.49	47.48
OJ_C2	0.28	3.02	1.25	0.12	9.69	27.76	46.99
OJ_1	0.33	3.27	1.46	0.16	11.69	46.52	70.09
OJ_2	0.38	3.78	2.00	0.19	13.01	51.05	77.06
OJ_3	0.50	3.64	1.39	0.18	13.83	45.75	73.39
OJ_4	0.55	3.85	1.90	0.19	17.84	50.31	85.97
OJ_5	0.33	3.03	1.36	0.13	11.67	38.36	61.28
OJ_6	0.34	3.31	1.77	0.18	12.96	42.57	67.88

OJ_7	0.39	3.01	1.35	0.12	13.67	37.55	64.59
OJ_8	0.44	3.44	1.71	0.18	14.61	42.50	71.80
KN_C1	0.22	1.65	0.13	0.00	9.78	0.38	19.94
KN_S	0.28	1.65	0.13	0.01	9.87	0.39	20.13
KN_C2	0.22	1.65	0.14	0.01	9.97	0.36	20.27
KN_1	0.28	1.69	0.26	0.01	11.04	4.76	26.85
KN_2	0.34	2.06	0.46	0.03	11.84	8.46	32.14
KN_3	0.39	1.77	0.27	0.05	11.91	5.11	28.84
KN_4	0.45	2.55	0.45	0.06	13.87	8.47	35.89
KN_5	0.27	1.67	0.25	0.01	9.87	3.63	23.38
KN_6	0.28	1.96	0.44	0.02	9.89	6.06	25.72
KN_7	0.34	1.80	0.26	0.03	11.60	4.54	27.67
KN_8	0.38	2.46	0.43	0.03	11.92	7.98	31.81
AS_C1	0.28	1.79	0.12	0.00	9.98	0.28	20.23
AS_S	0.34	1.82	0.20	0.01	9.98	0.30	20.27
AS_C2	0.28	1.80	0.15	0.00	9.91	0.26	20.09
AS_1	0.34	1.86	0.32	0.01	11.84	6.58	30.10
AS_2	0.39	1.94	0.44	0.10	13.65	9.28	36.37
AS_3	0.39	2.10	0.33	0.03	13.58	8.54	35.80
AS_4	0.43	2.45	0.54	0.17	15.09	9.54	39.73
AS_5	0.28	1.82	0.31	0.01	11.40	5.46	28.27
AS_6	0.32	1.92	0.44	0.04	13.46	7.85	35.13
AS_7	0.34	2.10	0.32	0.03	13.49	7.95	34.93
AS_8	0.39	2.42	0.52	0.13	13.96	8.54	36.23
1ºO_C1	0.61	2.77	0.51	0.03	19.59	22.46	61.65
1ºO_S	0.61	2.60	0.50	0.02	19.74	18.99	58.49
1ºO_C2	0.55	2.79	0.54	0.03	19.52	23.16	62.18
1ºO_1	0.67	2.86	0.74	0.04	21.99	36.14	80.12
1ºO_2	0.67	3.08	1.07	0.07	21.81	45.43	89.19
1ºO_3	0.66	2.93	0.86	0.07	23.58	41.72	88.88
1ºO_4	0.79	3.53	1.05	0.08	25.85	45.46	97.04
1ºO_5	0.62	2.83	0.74	0.03	21.59	29.24	72.43
1ºO_6	0.62	3.04	1.10	0.06	21.91	33.04	76.94
1ºO_7	0.62	2.87	0.82	0.05	21.67	36.27	79.51
1ºO_8	0.67	3.19	0.86	0.06	22.58	37.18	81.77
0.4ºO_C1	0.22	2.39	0.71	0.07	9.95	9.11	28.78
0.4ºO_S	0.22	2.41	0.72	0.06	9.98	10.45	29.94
0.4ºO_C2	0.22	2.36	0.73	0.06	9.80	10.90	30.42
0.4ºO_1	0.33	2.42	0.96	0.08	9.98	19.53	39.48
0.4ºO_2	0.39	3.69	1.08	0.10	11.88	31.92	55.68
0.4ºO_3	0.39	2.60	0.98	0.11	11.95	20.80	44.71
0.4ºO_4	0.45	3.81	1.20	0.12	15.79	29.81	61.37
0.4ºO_5	0.28	2.36	0.83	0.07	9.87	20.34	40.07
0.4ºO_6	0.33	3.63	1.00	0.09	11.79	30.43	54.06
0.4ºO_7	0.34	2.61	0.83	0.07	11.46	21.22	44.16

0.4ºO_8	0.39	3.75	1.16	0.11	13.73	29.86	57.31
SO_C	0.28	4.01	2.47	0.36	11.68	36.10	59.63
SO_1	0.28	4.41	2.55	0.39	13.85	52.04	79.59
SO_2	0.33	4.68	2.72	0.39	15.89	90.93	122.73
SO_3	0.34	4.52	2.59	0.41	15.89	44.52	76.34
SO_4	0.38	4.69	2.62	0.42	17.64	48.42	83.54
SOHO_C	0.17	3.17	0.97	0.12	3.98	24.46	32.41
SOHO_1	0.17	3.25	1.21	0.14	5.95	40.99	52.50
SOHO_2	0.22	3.96	1.45	0.15	5.88	44.71	56.63
SOHO_3	0.17	4.00	1.20	0.14	5.88	34.75	46.54
SOHO_4	0.22	3.92	1.36	0.16	7.85	41.89	57.56

PC_C1: Picual_Control 1; PC_S: Picual_Supplemented; PC_C2: Picual_Control 2; PC_1: Picual_Exp 1; PC_2: Picual_Exp 2; PC_3: Picual_Exp 3; PC_4: Picual_Exp 4; PC_5: Picual_Exp 5; PC_6: Picual_Exp 6; PC_7: Picual_Exp 7; PC_8: Picual_Exp 8; CC_C1: Cornicabra_Control 1; CC_S: Cornicabra_Supplemented; CC_C2: Cornicabra_Control 2; CC_1: Cornicabra_Exp 1; CC_2: Cornicabra_Exp 2; CC_3: Cornicabra_Exp 3; CC_4: Cornicabra_Exp 4; CC_5: Cornicabra_Exp 5; CC_6: Cornicabra_Exp 6; CC_7: Cornicabra_Exp 7; CC_8: Cornicabra_Exp 8; EP_C1: Empeltre_Control 1; EP_S: Empeltre_Supplemented; EP_C2: Empeltre_ Control 2; EP_1: Empeltre_Exp 1; EP_2: Empeltre_Exp 2; EP_3: Empeltre_Exp 3; EP_4: Empeltre_Exp 4; EP_5: Empeltre_Exp 5; EP_6: Empeltre_Exp 6; EP_7: Empeltre_Exp 7; EP_8: Empeltre_Exp 8; AQ_C1: Arbequina_Control 1; AQ_S: Arbequina_Supplemented; AQ_C2: Arbequina_ Control 2; AQ_1: Arbequina_Exp 1; AQ_2: Arbequina_Exp 2; AQ_3: Arbequina_Exp 3; AQ_4: Arbequina_Exp 4; AQ_5: Arbequina_Exp 5; AQ_6: Arbequina_Exp 6; AQ_7: Arbequina_Exp 7; AQ_8: Arbequina_Exp 8; HB_C1: Hojiblanca_Control 1; Hojiblanca_Supplemented; Hojiblanca_Control 2; HB_1: Hojiblanca_Exp 1; HB_2: Hojiblanca_Exp 2; HB_3: Hojiblanca_Exp 3; HB_4: Hojiblanca_Exp 4; HB_5: Hojiblanca_Exp 5; HB_6: Hojiblanca_Exp 6; HB_7: Hojiblanca_Exp 7; HB_8: Hojiblanca_Exp 8; MZ_C1: Manzanilla_ Control 1; MZ_S: Manzanilla_Supplemented; MZ_C2: Manzanilla_Control 2; MZ_1: Manzanilla_Exp 1; MZ_2: Manzanilla_Exp 2; MZ_3: Manzanilla_Exp 3; MZ_4: Manzanilla_Exp 4; MZ_5: Manzanilla_Exp 5; MZ_6: Manzanilla_Exp 6; MZ_7: Manzanilla_Exp 7; MZ_8: Manzanilla_Exp 8; RY_C1: Royuela_ Control 1; RY_S: Royuela_Supplemented; RY_C2: Royuela_ Control 2; RY_1: Royuela_Exp 1; RY_2: Royuela_Exp 2; RY_3: Royuela_Exp 3; RY_4: Royuela_Exp 4; RY_5: Royuela_Exp 5; RY_6: Royuela_Exp 6; RY_7: Royuela_Exp 7; RY_8: Royuela_Exp 8; OJ_C1: Pomace_Control 1; OJ_S: Pomace_ Supplemented; OJ_C2: Pomace_ Control 2; OJ_1: Pomace_Exp 1; OJ_2: Pomace_Exp 2; OJ_3: Pomace_Exp 3; OJ_4: Pomace_Exp 4; OJ_5: Pomace_Exp 5; OJ_6: Pomace_Exp 6; OJ_7: Pomace_Exp 7; OJ_8: Pomace_Exp 8; KN_C1: Koroneiki_Control 1; KN_S: Koroneiki_Supplemented; KN_C2: Koroneiki_Control 2; KN_1: Koroneiki_Exp 1; KN_2: Koroneiki_Exp 2; KN_3: Koroneiki_Exp 3; KN_4: Koroneiki_Exp 4; KN_5: Koroneiki_Exp 5; KN_6: Koroneiki_Exp 6; KN_7: Koroneiki_Exp 7; KN_8: Koroneiki_Exp 8; AS_C1: Arbosana_Control 1; AS_S: Arbosana_Supplemented; AS_C2: Arbosana_Control 2; AS_1: Arbosana_Exp 1; AS_2: Arbosana_Exp 2; AS_3: Arbosana_Exp 3; AS_4: Arbosana_Exp 4; AS_5: Arbosana_Exp 5; AS_6: Arbosana_Exp 6; AS_7: Arbosana_Exp 7; AS_8: Arbosana_Exp 8; 1°O_C1: Olive 1°_ Control 1; 1°O_S: Olive 1°_Supplemented; 1°O_C2: Olive 1°_ Control 2; 1°O_1: Olive 1°_Exp 1; 1°O_2: Olive 1°_Exp 2; 1°O_3: Olive 1°_Exp 3; 1°O_4: Olive 1°_Exp 4; 1°O_5: Olive 1°_Exp 5; 1°O_6: Olive 1°_Exp 6; 1°O_7: Olive 1°_Exp 7; 1°O_8: Olive 1°_Exp 8; 0.4°O_C1: Olive 0.4°_ Control 1; 0.4°O_S: Olive 0.4°_Supplemented; 0.4°O_C2: Olive 0.4°_ Control 2; 0.4°O_1: Olive 0.4°_Exp 1; 0.4°O_2: Olive 0.4°_Exp 2; 0.4°O_3: Olive 0.4°_Exp 3; 0.4°O_4: Olive 0.4°_Exp 4; 0.4°O_5: Olive 0.4°_Exp 5; 0.4°O_6: Olive 0.4°_Exp 6; 0.4°O_7: Olive 0.4°_Exp 7; 0.4°O_8: Olive 0.4°_Exp 8.

Sunflower oil_Control; SO_1: Sunflower oil_Exp 1; SO_2: Sunflower oil_Exp 2; SO_3: Sunflower oil_Exp 3; SO_4: Sunflower oil_Exp 4; SOHO_C: Sunflower oil_high oleic acid_Control; SOHO_1: Sunflower oil_ high oleic acid _Exp 1; SOHO_2: Sunflower oil_ high oleic acid_Exp 2; SOHO_3: Sunflower oil_ high oleic acid_Exp 3; SOHO_4: Sunflower oil_ high oleic acid_Exp 4. In addition, for the olive oil categories, Control 1 (used as the control for Experiments 1–4) refers to original, non-deep-fried olive oil. Supplemented oil refers to non-deep-fried olive oil that has been enriched with olive fruit extract, which is also used in the preparation of Control 2. Control 2 (used as the control for Experiments 5–8) is a mixture of Control 1 and the supplemented oil, resulting in a total polyphenol content of up to 650 mg/kg. Exp.1: olive oil deep-fried at 170 °C for 3 h without polyphenol supplementation, Exp.2: olive oil deep-fried at 170 °C for 6 h without polyphenol supplementation, Exp.3: olive oil deep-fried at 210 °C for 3 h without polyphenol supplementation, Exp.4: olive oil deep-fried at 210 °C for 6 h without polyphenol supplementation, Exp.5: olive oil deep-fried at 170 °C for 3 h with polyphenol supplementation, Exp.6: olive oil deep-fried at 170 °C for 6 h with polyphenol supplementation, Exp.7: olive oil deep-fried at 210 °C for 3 h with polyphenol supplementation, Exp.8: olive oil deep-fried at 210 °C for 6 h with polyphenol supplementation. Moreover, for sunflower oil categories: Control refers to original, non-deep-fried oil. Exp.1: oil deep-fried at 170 °C for 3 h, Exp.2: oil deep-fried at 170 °C for 6 h, Exp.3: oil deep-fried at 210 °C for 3 h, Exp.4: oil deep-fried at 210 °C for 6 h.
